# Supplementary material for: Does the Calcaneus Serve as Hypomochlion within the Lower Limb by a Myofascial Connection?—A Systematic Review
Source: Life (Basel). 2021 Jul 26;11(8):745. doi: 10.3390/life11080745 (PMC8398293; doi:10.3390/life11080745)
Supplement: Supplementary file 1 [file life-11-00745-s001.zip › life-1290010-supplementary.pdf]

Search results

Last search: July 2021

Databank: PubMed

”Achilles tendon” AND “Calcaneus” OR “Achilles tendon” AND “plantar fascia” OR “Calcaneus” AND “plantar fascia” as well as a search combination of “Achilles tendon” and “calcaneus” and “plantar fascia”

Total: 176 + 30

- Effectiveness of the Simultaneous Stretching of the Achilles Tendon and Plantar Fascia in Individuals With Plantar Fasciitis.
  - Engkananuwat P, Kanlayanaphotporn R, Purepong N.Engkananuwat P, et al. Foot Ankle Int. 2018 Jan;39(1):75-82. doi: 10.1177/1071100717732762. Epub 2017 Oct 6.Foot Ankle Int. 2018. PMID: 28985685

- Plantar Fasciitis.
  - Trojian T, Tucker AK.Trojian T, et al. Am Fam Physician. 2019 Jun 15;99(12):744-750.Am Fam Physician. 2019. PMID: 31194492

- Plantar Fasciitis.
  - Buchanan BK, Kushner D.Buchanan BK, et al. 2020 Jun 7. In: StatPearls [Internet]. Treasure Island (FL): StatPearls Publishing; 2021 Jan–.2020 Jun 7. In: StatPearls [Internet]. Treasure Island (FL): StatPearls Publishing; 2021 Jan–. PMID: 28613727 Free Books & Documents.

- The plantar calcaneal spur: a review of anatomy, histology, etiology and key associations.
  - Kirkpatrick J, Yassaie O, Mirjalili SA.Kirkpatrick J, et al. J Anat. 2017 Jun;230(6):743-751. doi: 10.1111/joa.12607. Epub 2017 Mar 29.J Anat. 2017. PMID: 28369929 Free PMC article.

- A Radiographic Study of Biomechanical Relationship between the Achilles Tendon and Plantar Fascia.
- Zhu G, Wang Z, Yuan C, Geng X, Zhang C, Huang J, Wang X, Ma X.Zhu G, et al. Biomed Res Int. 2020 Feb 18;2020:5319640. doi: 10.1155/2020/5319640. eCollection 2020.Biomed Res Int. 2020. PMID: 32149113

- Contributions of foot muscles and plantar fascia morphology to foot posture.
- Angin S, Mickle KJ, Nester CJ.Angin S, et al. Gait Posture. 2018 Mar;61:238-242. doi: 10.1016/j.gaitpost.2018.01.022. Epub 2018 Mar 20.Gait Posture. 2018. PMID: 29413791

- An ossifying bridge - on the structural continuity between the Achilles tendon and the plantar fascia.
- Zwirner J, Zhang M, Ondruschka B, Akita K, Hammer N.Zwirner J, et al. Sci Rep. 2020 Sep 3;10(1):14523. doi: 10.1038/s41598-020-71316-z.Sci Rep. 2020. PMID: 32884015

- Higher body mass index is associated with plantar fasciopathy/'plantar fasciitis': systematic review and meta-analysis of various clinical and imaging risk factors.
- van Leeuwen KD, Rogers J, Winzenberg T, van Middelkoop M.van Leeuwen KD, et al. Br J Sports Med. 2016 Aug;50(16):972-81. doi: 10.1136/bjsports-2015-094695. Epub 2015 Dec 7.Br J Sports Med. 2016. PMID: 26644427

- Influence of different knee and ankle ranges of motion on the elasticity of triceps surae muscles, Achilles tendon, and plantar fascia.
- Liu CL, Zhou JP, Sun PT, Chen BZ, Zhang J, Tang CZ, Zhang ZJ.Liu CL, et al. Sci Rep. 2020 Apr 20;10(1):6643. doi: 10.1038/s41598-020-63730-0.Sci Rep. 2020. PMID: 32313166

- Pes Cavus.
- Seaman TJ, Ball TA.Seaman TJ, et al. 2021 May 4. In: StatPearls [Internet]. Treasure Island (FL): StatPearls Publishing; 2021 Jan–.2021 May 4. In: StatPearls [Internet]. Treasure Island (FL): StatPearls Publishing; 2021 Jan–. PMID: 32310476 Free Books & Documents.

- Anatomy of the Achilles tendon and plantar fascia in relation to the calcaneus in various age groups.
- Snow SW, Bohne WH, DiCarlo E, Chang VK.Snow SW, et al. Foot Ankle Int. 1995 Jul;16(7):418-21. doi: 10.1177/107110079501600707.Foot Ankle Int. 1995. PMID: 7550955

- Effect of Achilles tendon loading on plantar fascia tension in the standing foot.
- Cheung JT, Zhang M, An KN.Cheung JT, et al. Clin Biomech (Bristol, Avon). 2006 Feb;21(2):194-203. doi: 10.1016/j.clinbiomech.2005.09.016. Epub 2005 Nov 8.Clin Biomech (Bristol, Avon). 2006. PMID: 16288943

- Classification of Calcaneal Spurs and Their Relationship With Plantar Fasciitis.
    - Zhou B, Zhou Y, Tao X, Yuan C, Tang K.Zhou B, et al. J Foot Ankle Surg. 2015 Jul-Aug;54(4):594-600. doi: 10.1053/j.jfas.2014.11.009. Epub 2015 Mar 11.J Foot Ankle Surg. 2015. PMID: 25771476

- Effectiveness of myofascial release in the management of plantar heel pain: a randomized controlled trial.
    - Ajimsha MS, Binsu D, Chithra S.Ajimsha MS, et al. Foot (Edinb). 2014 Jun;24(2):66-71. doi: 10.1016/j.foot.2014.03.005. Epub 2014 Mar 21.Foot (Edinb). 2014. PMID: 24703512

- Randomized controlled trial of calcaneal taping, sham taping, and plantar fascia stretching for the short-term management of plantar heel pain.
    - Hyland MR, Webber-Gaffney A, Cohen L, Lichtman PT.Hyland MR, et al. J Orthop Sports Phys Ther. 2006 Jun;36(6):364-71. doi: 10.2519/jospt.2006.2078.J Orthop Sports Phys Ther. 2006. PMID: 16776486

- Validity of enthesis ultrasound assessment in spondyloarthropathy.
    - de Miguel E, Cobo T, Muñoz-Fernández S, Naredo E, Usón J, Acebes JC, Andréu JL, Martín-Mola E.de Miguel E, et al. Ann Rheum Dis. 2009 Feb;68(2):169-74. doi: 10.1136/ard.2007.084251. Epub 2008 Apr 7.Ann Rheum Dis. 2009. PMID: 18390909

- On the morphological relations of the **Achilles tendon** and **plantar fascia** via the **calcaneus**: a cadaveric study.

- Singh A, Zwirner J, Templer F, Kieser D, Klima S, Hammer N.Singh A, et al. Sci Rep. 2021 Mar 16;11(1):5986. doi: 10.1038/s41598-021-85251-0.Sci Rep. 2021. PMID: 33727610

- Ultrasonography Features of the Plantar Fascia Complex in Patients with Chronic Non-Insertional Achilles Tendinopathy: A Case-Control Study.
  - Romero-Morales C, Martín-Llantino PJ, Calvo-Lobo C, López-López D, Sánchez-Gómez R, De-La-Cruz-Torres B, Rodríguez-Sanz D.Romero-Morales C, et al. Sensors (Basel). 2019 May 2;19(9):2052. doi: 10.3390/s19092052.Sensors (Basel). 2019. PMID: 31052554

- Ultrasonography Comparison of the Plantar Fascia and Tibialis Anterior in People With and Without Lateral Ankle Sprain: A Case-Control Study.
  - Romero-Morales C, López-López S, Bravo-Aguilar M, Cerezo-Téllez E, Benito-de Pedro M, López López D, Lobo CC.Romero-Morales C, et al. J Manipulative Physiol Ther. 2020 Oct;43(8):799-805. doi: 10.1016/j.jmpt.2019.11.004. Epub 2020 Jul 21.J Manipulative Physiol Ther. 2020. PMID: 32709515

- Anatomical variations of the plantar fascia's origin with respect to age and sex-an MRI based study.
  - Pękala PA, Kaythampillai L, Skinningsrud B, Loukas M, Walocha JA, Tomaszewski KA.Pękala PA, et al. Clin Anat. 2019 May;32(4):597-602. doi: 10.1002/ca.23342. Epub 2019 Apr 1.Clin Anat. 2019. PMID: 30701591

- THE MANAGEMENT OF PLANTAR FASCIITIS WITH A MUSCULOSKELETAL ULTRASOUND IMAGING GUIDED APPROACH FOR INSTRUMENT ASSISTED SOFT TISSUE MOBILIZATION IN A RUNNER: A CASE REPORT.
  - Sillevis R, Shamus E, Mouttet B.Sillevis R, et al. Int J Sports Phys Ther. 2020 Apr;15(2):274-286.Int J Sports Phys Ther. 2020. PMID: 32269861

- Effectiveness of myofascial trigger point manual therapy combined with a self-stretching protocol for the management of plantar heel pain: a randomized controlled trial.
  - Renan-Ordine R, Alburquerque-Sendín F, de Souza DP, Cleland JA, Fernández-de-Las-Peñas C.Renan-Ordine R, et al. J Orthop Sports Phys Ther. 2011 Feb;41(2):43-50. doi: 10.2519/jospt.2011.3504. Epub 2011 Jan 31.J Orthop Sports Phys Ther. 2011. PMID: 21285525

- Site- and sex-differences in morphological and mechanical properties of the plantar fascia: A supersonic shear imaging study.
- Shiotani H, Yamashita R, Mizokuchi T, Naito M, Kawakami Y.Shiotani H, et al. J Biomech. 2019 Mar 6;85:198-203. doi: 10.1016/j.jbiomech.2019.01.014. Epub 2019 Jan 14.J Biomech. 2019. PMID: 30665708

- Anatomy, Bony Pelvis and Lower Limb, Foot Fascia.
  - Bourne M, Talkad A, Varacallo M.Bourne M, et al. 2020 Aug 13. In: StatPearls [Internet]. Treasure Island (FL): StatPearls Publishing; 2021 Jan–.2020 Aug 13. In: StatPearls [Internet]. Treasure Island (FL): StatPearls Publishing; 2021 Jan–. PMID: 30252299 Free Books & Documents.

- Biomechanical analysis of minimally invasive crossing screw fixation for calcaneal fractures: Implications to early weight-bearing rehabilitation.
  - Zhang H, Lv ML, Liu Y, Sun W, Niu W, Wong DW, Ni M, Zhang M.Zhang H, et al. Clin Biomech (Bristol, Avon). 2020 Dec;80:105143. doi:

10.1016/j.clinbiomech.2020.105143. Epub 2020 Aug 15.Clin Biomech (Bristol, Avon). 2020. PMID: 32829234

- The Calcaneal Crescent in Patients With and Without Plantar Fasciitis: An Ankle MRI Study.
  - Finkenstaedt T, Siriwanarangsun P, Statum S, Biswas R, Anderson KE, Bae WC, Chung CB.Finkenstaedt T, et al. AJR Am J Roentgenol. 2018 Nov;211(5):1075-1082. doi: 10.2214/AJR.17.19399. Epub 2018 Aug 30.AJR Am J Roentgenol. 2018. PMID: 30160979

- MR Imaging Findings in Heel Pain.
  - Chang CD, Wu JS.Chang CD, et al. Magn Reson Imaging Clin N Am. 2017 Feb;25(1):79-93. doi: 10.1016/j.mric.2016.08.011.Magn Reson Imaging Clin N Am. 2017. PMID: 27888853

- A global approach for plantar fasciitis with extracorporeal shockwaves treatment.
  - Giordani F, Bernini A, Müller-Ehrenberg H, Stecco C, Masiero S.Giordani F, et al. Eur J Transl Myol. 2019 Sep 9;29(3):8372. doi: 10.4081/ejtm.2019.8372.
  - eCollection 2019 Aug 2.Eur J Transl Myol. 2019. PMID: 31579484

- Plantar fascia coronal length: a new parameter for plantar fascia assessment.
  - Sari AS, Demircay E, Cakmak G, Sahin MS, Tuncay IC, Altun S.Sari AS, et al. J Foot Ankle Surg. 2015 May-Jun;54(3):445-8. doi: 10.1053/j.jfas.2014.11.002. Epub 2014 Dec 5.J Foot Ankle Surg. 2015. PMID: 25488598

- Assessment of enthesis in patients with psoriatic arthritis and fibromyalgia using clinical examination and ultrasound.
  - Fiorenza A, Bonitta G, Gerratana E, Marino F, Sarzi-Puttini P, Salaffi F, Atzeni F.Fiorenza A, et al. Clin Exp Rheumatol. 2020 Jan-Feb;38 Suppl 123(1):31-39. Epub 2020 Feb 11.Clin Exp Rheumatol. 2020. PMID: 32116207

- Abnormal attachments between a plantar aponeurosis and calcaneus.
  - Kalniev MA, Krastev D, Krastev N, Vidinov K, Veltchev L, Mileva M.Kalniev MA, et al. Clujul Med. 2013;86(3):200-2. Epub 2013 Aug 5.Clujul Med. 2013. PMID: 26527947

- Calcaneal Avulsion Fractures: An Often Forgotten Diagnosis.
  - Yu SM, Yu JS.Yu SM, et al. AJR Am J Roentgenol. 2015 Nov;205(5):1061-7. doi: 10.2214/AJR.14.14190.AJR Am J Roentgenol. 2015. PMID: 26496554

- Interfascicular septum of the calcaneal tunnel and its relationship with the plantar nerves: A cadaveric study.
  - Cho TH, Kim SH, Won SY, O J, Kwon HJ, Won JY, Yang HM.Cho TH, et al. Clin Anat. 2019 Oct;32(7):877-882. doi: 10.1002/ca.23381. Epub 2019 Apr 15.Clin Anat. 2019. PMID: 30945342

- Plantar Medial Avulsion Fragment Associated With Tongue-Type Calcaneus Fractures.
- Adams MR, Koury KL, Mistry JB, Braaksma W, Hwang JS, Firoozabadi R.Adams MR, et al. Foot Ankle Int. 2019 Jun;40(6):634-640. doi: 10.1177/1071100719830948. Epub 2019 Mar 6.Foot Ankle Int. 2019. PMID: 30841752

- Ultrasound of entheses in ankylosing spondylitis patients: The importance of the calcaneal and quadriceps entheses for differentiating patients from healthy individuals.
    - Ishida SN, Furtado RNV, Rosenfeld A, Proglhof JEP, Estrela GBQ, Natour J.Ishida SN, et al. Clinics (Sao Paulo). 2019;74:e727. doi: 10.6061/clinics/2019/e727. Epub 2019 Apr 8.Clinics (Sao Paulo). 2019. PMID: 30970118

- [Plantar fascia release and decompression of the first branch of the lateral plantar nerve (Baxter's nerve)].
- Arbab D, Bouillon B, Lüring C, Störmann S, Gutteck N.Arbab D, et al. Oper Orthop Traumatol. 2021 Jul 13. doi: 10.1007/s00064-021-00720-z. Online ahead of print.Oper Orthop Traumatol. 2021. PMID: 34255092

- Medial Soft-Tissue Release for Lateralising Calcaneal Osteotomy: A Cadaveric Study.
    - Dash KK, Bradley R, Stavrakakis I, Shah K.Dash KK, et al. Indian J Orthop. 2020 Jan 13;54(1):49-54. doi: 10.1007/s43465-019-00017-1. eCollection 2020 Feb.Indian J Orthop. 2020. PMID: 32257016

- Radiological characteristics of the calcaneal spurs in psoriatic arthritis.
    - Gladman DD, Abufayyah M, Salonen D, Thavaneswaran A, Chandran V.Gladman DD, et al. Clin Exp Rheumatol. 2014 May-Jun;32(3):401-3. Epub 2014 May 12.Clin Exp Rheumatol. 2014. PMID: 24850064

- Percutaneous release of the plantar fascia. New surgical procedure.
    - Oliva F, Piccirilli E, Tarantino U, Maffulli N.Oliva F, et al. Muscles Ligaments Tendons J. 2017 Sep 18;7(2):338-340. doi: 10.11138/mltj/2017.7.2.338. eCollection 2017 Apr-Jun.Muscles Ligaments Tendons J. 2017. PMID: 29264346

- Finite element analysis of plantar fascia during walking: a quasi-static simulation.
    - Chen YN, Chang CW, Li CT, Chang CH, Lin CF.Chen YN, et al. Foot Ankle Int. 2015 Jan;36(1):90-7. doi: 10.1177/1071100714549189. Epub 2014 Sep 4.Foot Ankle Int. 2015. PMID: 25189539

- Heel spur formation and the subcalcaneal enthesis of the plantar fascia.
    - Kumai T, Benjamin M.Kumai T, et al. J Rheumatol. 2002 Sep;29(9):1957-64.J Rheumatol. 2002. PMID: 12233893

- Endoscopic plantar fascia release, calcaneal drilling and calcaneal spur removal for management of painful heel syndrome.
    - El Shazly O, El Beltagy A.El Shazly O, et al. Foot (Edinb). 2010 Dec;20(4):121-5. doi: 10.1016/j.foot.2010.09.004.Foot (Edinb). 2010. PMID: 20926285

- The anatomical footprint of the Achilles tendon: a cadaveric study.
    - Ballal MS, Walker CR, Molloy AP.Ballal MS, et al. Bone Joint J. 2014 Oct;96-B(10):1344-8. doi: 10.1302/0301-620X.96B10.33771.Bone Joint J. 2014. PMID: 25274919

- Calcaneal attachment of the plantar fascia: MR findings in asymptomatic volunteers.
    - Ehrmann C, Maier M, Mengiardi B, Pfirrmann CW, Sutter R.Ehrmann C, et al. Radiology. 2014 Sep;272(3):807-14. doi: 10.1148/radiol.14131410. Epub 2014 May 7.Radiology. 2014. PMID: 24814176

- Mid-Sole Release of the Plantar Fascia Combined With Percutaneous Drilling of the Calcaneus for Treatment of Resistant Heel Pain.
    - Rizk AS, Kandel WA, Tabl EAE, Kandil MI.Rizk AS, et al. Foot Ankle Int. 2017 Nov;38(11):1271-1277. doi: 10.1177/1071100717723131. Epub 2017 Sep 9.Foot Ankle Int. 2017. PMID: 28891313

- Effect of gender, age and anthropometric variables on plantar fascia thickness at different locations in asymptomatic subjects.
    - Pascual Huerta J, Alarcón García JM.Pascual Huerta J, et al. Eur J Radiol. 2007 Jun;62(3):449-53. doi: 10.1016/j.ejrad.2007.01.002. Epub 2007 Feb 5.Eur J Radiol. 2007. PMID: 17284352

- The variability of the Achilles tendon insertion: a cadaveric examination.
    - Kim PJ, Richey JM, Wissman LR, Steinberg JS.Kim PJ, et al. J Foot Ankle Surg. 2010 Sep-Oct;49(5):417-20. doi: 10.1053/j.jfas.2010.05.002. Epub 2010 Jun 25.J Foot Ankle Surg. 2010. PMID: 20579910

- Development of the human Achilles tendon enthesis organ.
    - Shaw HM, Vázquez OT, McGonagle D, Bydder G, Santer RM, Benjamin M.Shaw HM, et al. J Anat. 2008 Dec;213(6):718-24. doi: 10.1111/j.1469-7580.2008.00997.x.J Anat. 2008. PMID: 19094187

- The role of biomechanical factors and HLA-B27 in magnetic resonance imaging-determined bone changes in plantar fascia enthesopathy.
    - McGonagle D, Marzo-Ortega H, O'Connor P, Gibbon W, Pease C, Reece R, Emery P.McGonagle D, et al. Arthritis Rheum. 2002 Feb;46(2):489-93. doi: 10.1002/art.10125.Arthritis Rheum. 2002. PMID: 11840452

- Shear Wave Elastography (SWE) for the Evaluation of Patients with Plantar Fasciitis.
    - Gatz M, Bejder L, Quack V, Schrading S, Dirrichs T, Tingart M, Kuhl C, Betsch M.Gatz M, et al. Acad Radiol. 2020 Mar;27(3):363-370. doi: 10.1016/j.acra.2019.04.009. Epub 2019 May 30.Acad Radiol. 2020. PMID: 31153782
- Finite element analysis of the foot: Stress and displacement shielding.
    - Filardi V.Filardi V. J Orthop. 2018 Sep 6;15(4):974-979. doi: 10.1016/j.jor.2018.08.037. eCollection 2018 Dec.J Orthop. 2018. PMID: 30224853

- Plantar Medial Avulsion Fracture of the Calcaneus With Acute Tarsal Tunnel: Case Report and Technique Tip.
    - Walley KC, Johns WL, Jackson JB, Gonzalez TA.Walley KC, et al. Foot Ankle Int. 2020 Aug;41(8):1002-1006. doi: 10.1177/1071100720924379. Epub 2020 Jul 7.Foot Ankle Int. 2020. PMID: 32635754

- Plantar Fascia Release Through a Single Lateral Incision in the Operative Management of a Cavovarus Foot: A Cadaver Model Analysis of the Operative Technique.
    - Kiskaddon EM, Meeks BD, Roberts JG, Laughlin RT.Kiskaddon EM, et al. J Foot Ankle Surg. 2018 Jul-Aug;57(4):681-684. doi: 10.1053/j.jfas.2017.11.042. Epub 2018 Apr 4.J Foot Ankle Surg. 2018. PMID: 29627135

- Topographical Pressure Pain Sensitivity Maps of the Feet Reveal Bilateral Pain Sensitivity in Patients With Unilateral Plantar Heel Pain.

- Ríos-León M, Ortega-Santiago R, Madeleine P, Fernández-de-Las-Peñas C, Plaza-Manzano G.Ríos-León M, et al. J Orthop Sports Phys Ther. 2019 Sep;49(9):640-646. doi: 10.2519/jospt.2019.8813. Epub 2019 Mar 26.J Orthop Sports Phys Ther. 2019. PMID: 30913970

- Targeting the Plantar Fascia for Corticosteroid Injection.
    - Salvi AE.Salvi AE. J Foot Ankle Surg. 2015 Jul-Aug;54(4):683-5. doi: 10.1053/j.jfas.2014.10.011.J Foot Ankle Surg. 2015. PMID: 25432461

- [Assessment of plantar fasciitis using shear wave elastography].
    - Zhang L, Wan W, Zhang L, Xiao H, Luo Y, Fei X, Zheng Z, Tang P.Zhang L, et al. Nan Fang Yi Ke Da Xue Xue Bao. 2014 Feb;34(2):206-9.Nan Fang Yi Ke Da Xue Xue Bao. 2014. PMID: 24589597 Chinese.

- Development of a Preliminary Ultrasonographic Enthesitis Score in Psoriatic Arthritis - GRAPPA Ultrasound Working Group.
- Tom S, Zhong Y, Cook R, Aydin SZ, Kaeley G, Eder L.Tom S, et al. J Rheumatol. 2019 Apr;46(4):384-390. doi: 10.3899/jrheum.171465. Epub 2018 Oct 15.J Rheumatol. 2019. PMID: 30323008

- Acupotomy for calcaneodynia: A systematic review protocol.
    - Shen Y, Zhou Q, Qiu Z, Jia Y, Li S.Shen Y, et al. Medicine (Baltimore). 2018 Apr;97(14):e0143. doi: 10.1097/MD.0000000000010143.Medicine (Baltimore). 2018. PMID: 29620626

- Subcalcaneal bursitis with plantar fasciitis treated by arthroscopy.
    - Yamakado K.Yamakado K. Arthrosc Tech. 2013 Apr 18;2(2):e135-9. doi: 10.1016/j.eats.2013.01.003. Print 2013 May.Arthrosc Tech. 2013. PMID: 23875139

- Effect of calcaneal osteotomy and lateral column lengthening on the plantar fascia: a biomechanical investigation.
    - Horton GA, Myerson MS, Parks BG, Park YW.Horton GA, et al. Foot Ankle Int. 1998 Jun;19(6):370-3. doi: 10.1177/107110079801900605.Foot Ankle Int. 1998. PMID: 9677079

- Observations on the fibrous retinacula of the heel pad.
    - Snow SW, Bohne WH.Snow SW, et al. Foot Ankle Int. 2006 Aug;27(8):632-5. doi: 10.1177/107110070602700812.Foot Ankle Int. 2006. PMID: 16919218

- Effect of foot orthoses as treatment for plantar fasciitis or heel pain.
    - Anderson J, Stanek J.Anderson J, et al. J Sport Rehabil. 2013 May;22(2):130-6. doi: 10.1123/jsr.22.2.130. Epub 2012 Oct 2.J Sport Rehabil. 2013. PMID: 23037146

- Does the minimally invasive complete plantar fasciotomy result in deformity of the Plantar arch? A prospective study.
    - De Prado M, Cuervas-Mons M, De Prado V, Golanó P, Vaquero J.De Prado M, et al. Foot Ankle Surg. 2020 Apr;26(3):347-353. doi: 10.1016/j.fas.2019.04.010. Epub 2019 Apr 27.Foot Ankle Surg. 2020. PMID: 31113726

- Sonographically guided deep plantar fascia injections: where does the injectate go?

  - Maida E, Presley JC, Murthy N, Pawlina W, Smith J.Maida E, et al. J Ultrasound Med. 2013 Aug;32(8):1451-9. doi: 10.7863/ultra.32.8.1451.J Ultrasound Med. 2013. PMID: 23887956

- Primary Care Management of Plantar Fasciitis.
  - Melvin TJ, Tankersley ZJ, Qazi ZN, Jasko JJ, Odono R, Shuler FD.Melvin TJ, et al. W V Med J. 2015 Nov-Dec;111(6):28-32.W V Med J. 2015. PMID: 26665894

- [Applied anatomy of medial plantar artery combined flaps and repairing heel and adjacent vast soft tissue defects].
  - Wei ZR, Wang DL, Wang YM, Sun GF, Tang XJ, Wang B.Wei ZR, et al. Zhonghua Yi Xue Za Zhi. 2009 Jun 9;89(22):1553-7.Zhonghua Yi Xue Za Zhi. 2009. PMID: 19953884 Chinese.

- Deep fascia of the foot. Anatomical and clinical considerations.
  - Mitchell IR, Meyer C, Krueger WA.Mitchell IR, et al. J Am Podiatr Med Assoc. 1991 Jul;81(7):373-8. doi: 10.7547/87507315-81-7-373.J Am Podiatr Med Assoc. 1991. PMID: 1941581

- [MRI of plantar fasciitis].
  - Steinborn M, Heuck A, Maier M, Schnarkowski P, Scheidler J, Reiser M.Steinborn M, et al. Rofo. 1999 Jan;170(1):41-6. doi: 10.1055/s-2007-1011005.Rofo. 1999. PMID: 10071643

- Calcaneal osteotomy for the treatment of plantar fasciitis.
  - Miyamoto W, Takao M, Uchio Y.Miyamoto W, et al. Arch Orthop Trauma Surg. 2010 Feb;130(2):151-4. doi: 10.1007/s00402-009-0879-8. Epub 2009 Apr 21.Arch Orthop Trauma Surg. 2010. PMID: 19381659

- Three-dimensional reconstructions of the Achilles tendon insertion in man.
  - Milz S, Rufai A, Buettner A, Putz R, Ralphs JR, Benjamin M.Milz S, et al. J Anat. 2002 Feb;200(Pt 2):145-52. doi: 10.1046/j.0021-8782.2001.00016.x.J Anat. 2002. PMID: 11895112

- Towards patient-specific medializing calcaneal osteotomy for adult flatfoot: a finite element study.
  - Wang Z, Kido M, Imai K, Ikoma K, Hirai S.Wang Z, et al. Comput Methods Biomech Biomed Engin. 2018 Mar;21(4):332-343. doi: 10.1080/10255842.2018.1452202. Epub 2018 Mar 15.Comput Methods Biomech Biomed Engin. 2018. PMID: 29544347

- Comparison of Ultrasound-Guided Local Ozone (O2-O3) Injection vs Corticosteroid Injection in the Treatment of Chronic Plantar Fasciitis: A Randomized Clinical Trial.
  - Babaei-Ghazani A, Karimi N, Forogh B, Madani SP, Ebadi S, Fadavi HR, Sobhani-Eraghi A, Emami Razavi SZ, Raeissadat SA, Eftekharsadat B.Babaei-Ghazani A, et al. Pain Med. 2019 Feb 1;20(2):314-322. doi: 10.1093/pm/pny066.Pain Med. 2019. PMID: 29868796 Clinical Trial.

- The present status of the problem of pes cavus.
  - Dwyer FC.Dwyer FC. Clin Orthop Relat Res. 1975 Jan-Feb;(106):254-75. doi: 10.1097/00003086-197501000-00038.Clin Orthop Relat Res. 1975. PMID: 1092504

- Subcalcaneal heel pain.

    - Karr SD.Karr SD. Orthop Clin North Am. 1994 Jan;25(1):161-75.Orthop Clin North Am. 1994. PMID: 8290225

- Passive hallux adduction decreases lateral plantar artery blood flow: a preliminary study of the potential influence of narrow toe box shoes.
    - Jacobs JL, Ridge ST, Bruening DA, Brewerton KA, Gifford JR, Hoopes DM, Johnson AW.Jacobs JL, et al. J Foot Ankle Res. 2019 Nov 4;12:50. doi: 10.1186/s13047-019-0361-y. eCollection 2019.J Foot Ankle Res. 2019. PMID: 31700547

- [Surgical treatment of rupture of the plantar fascia].
    - Christel P, Rigal S, Poux D, Roger B, Witvoët J.Christel P, et al. Rev Chir Orthop Reparatrice Appar Mot. 1993;79(3):218-25.Rev Chir Orthop Reparatrice Appar Mot. 1993. PMID: 8122010

- Endoscopic plantar fasciotomy.
    - Barrett SL.Barrett SL. Clin Podiatr Med Surg. 1994 Jul;11(3):469-81.Clin Podiatr Med Surg. 1994. PMID: 7954212

- Plantar fasciitis: sonographic evaluation.
    - Cardinal E, Chhem RK, Beauregard CG, Aubin B, Pelletier M.Cardinal E, et al. Radiology. 1996 Oct;201(1):257-9. doi: 10.1148/radiology.201.1.8816554.Radiology. 1996. PMID: 8816554

- Endoscopic surgery for plantar fasciitis: application of a deep-fascial approach.
    - Komatsu F, Takao M, Innami K, Miyamoto W, Matsushita T.Komatsu F, et al. Arthroscopy. 2011 Aug;27(8):1105-9. doi: 10.1016/j.arthro.2011.02.037. Epub 2011 Jun 24.Arthroscopy. 2011. PMID: 21704466

- Anatomy of medial plantar superficial branch artery perforators: Facilitation of medial plantar superficial branch artery perforator (MPAP) flap harvesting and design for finger pulp reconstruction.
    - Lohasammakul S, Turbpaiboon C, Chaiyasate K, Tatsanavivat P, Chompoopong S, Roham A, Ratanalekha R, Aojanepong C.Lohasammakul S, et al. Microsurgery. 2018 Jul;38(5):536-543. doi: 10.1002/micr.30321. Epub 2018 Mar 25.Microsurgery. 2018. PMID: 29575166

- The contribution of the medial calcaneal osteotomy to the correction of flatfoot deformities.
    - Nyska M, Parks BG, Chu IT, Myerson MS.Nyska M, et al. Foot Ankle Int. 2001 Apr;22(4):278-82. doi: 10.1177/107110070102200402.Foot Ankle Int. 2001. PMID: 11354439

- Cartilage Stiffness Effect on Foot Biomechanics of Chinese Bound Foot: A Finite Element Analysis.
    - Zhang Y, Awrejcewicz J, Baker JS, Gu Y.Zhang Y, et al. Front Physiol. 2018 Oct 11;9:1434. doi: 10.3389/fphys.2018.01434. eCollection 2018.Front Physiol. 2018. PMID: 30364272

- Magnetic resonance imaging in plantar heel pain.
- Chimutengwende-Gordon M, O'Donnell P, Singh D.Chimutengwende-Gordon M, et al. Foot Ankle Int. 2010 Oct;31(10):865-70. doi: 10.3113/FAI.2010.0865.Foot Ankle Int. 2010. PMID: 20964964

- Endoscopic plantar fascia release: an anatomical study.

- Hofmeister EP, Elliott MJ, Juliano PJ.Hofmeister EP, et al. Foot Ankle Int. 1995 Nov;16(11):719-23. doi: 10.1177/107110079501601109.Foot Ankle Int. 1995. PMID: 8589812

- Widespread Pressure Pain Hypersensitivity in Musculoskeletal and Nerve Trunk Areas as a Sign of Altered Nociceptive Processing in Unilateral Plantar Heel Pain.
  - Plaza-Manzano G, Ríos-León M, Martín-Casas P, Arendt-Nielsen L, Fernández-de-Las-Peñas C, Ortega-Santiago R.Plaza-Manzano G, et al. J Pain. 2019 Jan;20(1):60-67. doi: 10.1016/j.jpain.2018.08.001. Epub 2018 Aug 16.J Pain. 2019. PMID: 30121357

- Sonographic evaluation of plantar fasciitis and relation to body mass index.
  - Ozdemir H, Yilmaz E, Murat A, Karakurt L, Poyraz AK, Ogur E.Ozdemir H, et al. Eur J Radiol. 2005 Jun;54(3):443-7. doi: 10.1016/j.ejrad.2004.09.004.Eur J Radiol. 2005. PMID: 15899349

- Bilateral calcaneal insufficiency fractures due to chronic carbamazepine use for trigeminal neuralgia: A case report.
  - Kaya O, Hurel C, Gumussuyu G, Kose O.Kaya O, et al. Niger J Clin Pract. 2020 Apr;23(4):574-576. doi: 10.4103/njcp.njcp_515_18.Niger J Clin Pract. 2020. PMID: 32246668

- Anatomic basis of plantar flap design.
  - Hidalgo DA, Shaw WW.Hidalgo DA, et al. Plast Reconstr Surg. 1986 Nov;78(5):627-36.Plast Reconstr Surg. 1986. PMID: 3763749

- Magnetic resonance imaging findings of chronic plantar fasciitis before and after extracorporeal shock wave therapy.
  - Maki M, Ikoma K, Kido M, Hara Y, Sawada K, Ohashi S, Kubo T.Maki M, et al. Foot (Edinb). 2017 Dec;33:25-28. doi: 10.1016/j.foot.2017.10.002. Epub 2017 Oct 26.Foot (Edinb). 2017. PMID: 29126038

- Reconstruction with tenodesis in an adult flatfoot model. A biomechanical evaluation of four methods.
  - Thordarson DB, Schmotzer H, Chon J.Thordarson DB, et al. J Bone Joint Surg Am. 1995 Oct;77(10):1557-64. doi: 10.2106/00004623-199510000-00011.J Bone Joint Surg Am. 1995. PMID: 7593065

- Three-dimensional printed calcaneal prosthesis following total calcanectomy.
  - Imanishi J, Choong PF.Imanishi J, et al. Int J Surg Case Rep. 2015;10:83-7. doi: 10.1016/j.ijscr.2015.02.037. Epub 2015 Mar 10.Int J Surg Case Rep. 2015. PMID: 25827294

- Injuries observed in minimalist runners.
  - Salzler MJ, Bluman EM, Noonan S, Chiodo CP, de Asla RJ.Salzler MJ, et al. Foot Ankle Int. 2012 Apr;33(4):262-6. doi: 10.3113/FAI.2012.0262.Foot Ankle Int. 2012. PMID: 22735197

- Heel pain--operative results.
  - Baxter DE, Thigpen CM.Baxter DE, et al. Foot Ankle. 1984 Jul-Aug;5(1):16-25. doi: 10.1177/107110078400500103.Foot Ankle. 1984. PMID: 6479759

- 3D printing individualized heel cup for improving the self-reported pain of plantar fasciitis.

    - Li L, Yang L, Yu F, Shi J, Zhu L, Yang X, Teng H, Wang X, Jiang Q.Li L, et al. J Transl Med. 2018 Jun 18;16(1):167. doi: 10.1186/s12967-018-1547-y.J Transl Med. 2018. PMID: 29914501

- Detection of enthesitis in children with enthesitis-related arthritis: dolorimetry compared to ultrasonography.
    - Weiss PF, Chauvin NA, Klink AJ, Localio R, Feudtner C, Jaramillo D, Colbert RA, Sherry DD, Keren R.Weiss PF, et al. Arthritis Rheumatol. 2014 Jan;66(1):218-27. doi: 10.1002/art.38197.Arthritis Rheumatol. 2014. PMID: 24449586

- Three-dimensional finite element analysis of the foot during standing--a material sensitivity study.
    - Cheung JT, Zhang M, Leung AK, Fan YB.Cheung JT, et al. J Biomech. 2005 May;38(5):1045-54. doi: 10.1016/j.jbiomech.2004.05.035.J Biomech. 2005. PMID: 15797586

- Ultrasound detection of entheseal insertions in the foot of patients with spondyloarthropathy.
    - Borman P, Koparal S, Babaoğlu S, Bodur H.Borman P, et al. Clin Rheumatol. 2006 May;25(3):373-7. doi: 10.1007/s10067-005-0036-x. Epub 2005 Nov 1.Clin Rheumatol. 2006. PMID: 16261286

- Biomechanical consequences of plantar fascial release or rupture during gait. Part II: alterations in forefoot loading.
    - Sharkey NA, Donahue SW, Ferris L.Sharkey NA, et al. Foot Ankle Int. 1999 Feb;20(2):86-96. doi: 10.1177/107110079902000204.Foot Ankle Int. 1999. PMID: 10063976

- Clinical Enthesitis in a Prospective Longitudinal Psoriatic Arthritis Cohort: Incidence, Prevalence, Characteristics, and Outcome.
    - Polachek A, Li S, Chandran V, Gladman DD.Polachek A, et al. Arthritis Care Res (Hoboken). 2017 Nov;69(11):1685-1691. doi: 10.1002/acr.23174. Epub 2017 Sep 21.Arthritis Care Res (Hoboken). 2017. PMID: 27998023

- Patient specific computational models to optimize surgical correction for flatfoot deformity.
- Smith BA, Adelaar RS, Wayne JS.Smith BA, et al. J Orthop Res. 2017 Jul;35(7):1523-1531. doi: 10.1002/jor.23399. Epub 2016 Sep 19.J Orthop Res. 2017. PMID: 27556250

- Biomechanical consequences of sequential plantar fascia release.
    - Murphy GA, Pneumaticos SG, Kamaric E, Noble PC, Trevino SG, Baxter DE.Murphy GA, et al. Foot Ankle Int. 1998 Mar;19(3):149-52. doi: 10.1177/107110079801900306.Foot Ankle Int. 1998. PMID: 9542985

- Effect of the calcaneal medializing osteotomy on soft tissues supporting the plantar arch: A computational study.
    - Larrainzar-Garijo R, Cifuentes de la Portilla C, Gutiérrez-Narvarte B, Díez-Nicolás E, Bayod J.Larrainzar-Garijo R, et al. Rev Esp Cir Ortop Traumatol (Engl Ed). 2019 Mar-Apr;63(2):155-163. doi: 10.1016/j.recot.2018.04.003. Epub 2018 Jun 12.Rev Esp Cir Ortop Traumatol (Engl Ed). 2019. PMID: 29907523

- A study of calcaneal enthesophytes (spurs) in Indian population.

- Kullar JS, Randhawa GK, Kullar KK.Kullar JS, et al. Int J Appl Basic Med Res. 2014 Sep;4(Suppl 1):S13-6. doi: 10.4103/2229-516X.140709.Int J Appl Basic Med Res. 2014. PMID: 25298934

- Using an optimization approach to design an insole for lowering plantar fascia stress--a finite element study.
  - Hsu YC, Gung YW, Shih SL, Feng CK, Wei SH, Yu CH, Chen CS.Hsu YC, et al. Ann Biomed Eng. 2008 Aug;36(8):1345-52. doi: 10.1007/s10439-008-9516-x. Epub 2008 May 15.Ann Biomed Eng. 2008. PMID: 18481179

- Plantar Fasciitis: Diagnosis and Conservative Management.
  - Gill LH.Gill LH. J Am Acad Orthop Surg. 1997 Mar;5(2):109-117. doi: 10.5435/00124635-199703000-00006.J Am Acad Orthop Surg. 1997. PMID: 10797213

- A biomechanical approach to the prevention, treatment and rehabilitation of plantar fasciitis.
  - Chandler TJ, Kibler WB.Chandler TJ, et al. Sports Med. 1993 May;15(5):344-52. doi: 10.2165/00007256-199315050-00006.Sports Med. 1993. PMID: 8100639

- Analysis of Pressure Pain Hypersensitivity, Ultrasound Image, and Quality of Life in Patients with Chronic Plantar Pain: A Preliminary Study.
  - Fernández-Lao C, Galiano-Castillo N, Cantarero-Villanueva I, Martín-Martín L, Prados-Olleta N, Arroyo-Morales M.Fernández-Lao C, et al. Pain Med. 2016 Aug;17(8):1530-41. doi: 10.1093/pm/pnv022. Epub 2016 Jan 6.Pain Med. 2016. PMID: 26814301

- Endoscopic Debridement for Treatment of Chronic Plantar Fasciitis: An Innovative Technique and Prospective Study of 46 Consecutive Patients.
  - Cottom JM, Maker JM, Richardson P, Baker JS.Cottom JM, et al. J Foot Ankle Surg. 2016 Jul-Aug;55(4):748-52. doi: 10.1053/j.jfas.2016.02.005. Epub 2016 Apr 5.J Foot Ankle Surg. 2016. PMID: 27066869

- Tibiotalocalcaneal arthrodesis: anatomic and technical considerations.
  - McGarvey WC, Trevino SG, Baxter DE, Noble PC, Schon LC.McGarvey WC, et al. Foot Ankle Int. 1998 Jun;19(6):363-9. doi: 10.1177/107110079801900604.Foot Ankle Int. 1998. PMID: 9677078

- Comparison of Extraosseous Talotarsal Stabilization Implants in a Stage II Adult-Acquired Flatfoot Model: A Finite Element Analysis.
  - Xu J, Ma X, Wang D, Lu W, Zhu W, Ouyang K, Liu H, Li H, Jiang L.Xu J, et al. J Foot Ankle Surg. 2017 Sep-Oct;56(5):1058-1064. doi: 10.1053/j.jfas.2017.05.009. Epub 2017 Jun 13.J Foot Ankle Surg. 2017. PMID: 28623061

- Elastofibroma in the Rearfoot: A Case Report of a Rare Soft Tissue Tumor.
  - Pirak J, Brandeisky JA, Simon P, Khaladj M.Pirak J, et al. J Foot Ankle Surg. 2020 May-Jun;59(3):587-589. doi: 10.1053/j.jfas.2019.09.021.J Foot Ankle Surg. 2020. PMID: 32354514

- Effect of calcaneal osteotomy and plantar fasciotomy on arch configuration in a flatfoot model.

    - Thordarson DB, Hedman T, Lundquist D, Reisch R.Thordarson DB, et al. Foot Ankle Int. 1998 Jun;19(6):374-8. doi: 10.1177/107110079801900606.Foot Ankle Int. 1998. PMID: 9677080

- Indication, surgical technique and results of endoscopic fascial release in plantar fasciitis (E FRPF).
    - Jerosch J, Schunck J, Liebsch D, Filler T.Jerosch J, et al. Knee Surg Sports Traumatol Arthrosc. 2004 Sep;12(5):471-7. doi: 10.1007/s00167-004-0496-6. Epub 2004 Apr 14.Knee Surg Sports Traumatol Arthrosc. 2004. PMID: 15088083

- Pathophysiology of Charcot-Marie-Tooth disease.
    - Mann RA, Missirian J.Mann RA, et al. Clin Orthop Relat Res. 1988 Sep;(234):221-8.Clin Orthop Relat Res. 1988. PMID: 3409580

- Heel pain syndrome: electrodiagnostic support for nerve entrapment.
    - Schon LC, Glennon TP, Baxter DE.Schon LC, et al. Foot Ankle. 1993 Mar-Apr;14(3):129-35. doi: 10.1177/107110079301400304.Foot Ankle. 1993. PMID: 8491426

- Clinical significance of musculoskeletal finite element model of the second and the fifth foot ray with metatarsal cavities and calcaneal sinus.
    - Wu L, Zhong S, Zheng R, Qu J, Ding Z, Tang M, Wang X, Hong J, Zheng X, Wang X.Wu L, et al. Surg Radiol Anat. 2007 Oct;29(7):561-7. doi: 10.1007/s00276-007-0231-3. Epub 2007 Jul 10.Surg Radiol Anat. 2007. PMID: 17619812

- Plantar fasciitis. Etiology, treatment, surgical results, and review of the literature.
    - Schepsis AA, Leach RE, Gorzyca J.Schepsis AA, et al. Clin Orthop Relat Res. 1991 May;(266):185-96.Clin Orthop Relat Res. 1991. PMID: 2019049

- Magnetic resonance imaging of plantar fasciitis and other causes of heel pain.
    - Kier R.Kier R. Magn Reson Imaging Clin N Am. 1994 Feb;2(1):97-107.Magn Reson Imaging Clin N Am. 1994. PMID: 7584243

- Surgical treatment of plantar fasciitis.
    - Lester DK, Buchanan JR.Lester DK, et al. Clin Orthop Relat Res. 1984 Jun;(186):202-4.Clin Orthop Relat Res. 1984. PMID: 6723144

- Operative treatment of plantar fasciitis.
    - Kulthanan T.Kulthanan T. J Med Assoc Thai. 1992 Jun;75(6):337-40.J Med Assoc Thai. 1992. PMID: 1487681

- Shock wave therapy for chronic proximal plantar fasciitis.
    - Ogden JA, Alvarez R, Levitt R, Cross GL, Marlow M.Ogden JA, et al. Clin Orthop Relat Res. 2001 Jun;(387):47-59. doi: 10.1097/00003086-200106000-00007.Clin Orthop Relat Res. 2001. PMID: 11400894

- Real-time subject-specific monitoring of internal deformations and stresses in the soft tissues of the foot: a new approach in gait analysis.
    - Yarnitzky G, Yizhar Z, Gefen A.Yarnitzky G, et al. J Biomech. 2006;39(14):2673-89. doi: 10.1016/j.jbiomech.2005.08.021. Epub 2005 Oct 5.J Biomech. 2006. PMID: 16212969

- Calcaneal Osteotomy Safe Zone to Prevent Neurological Damage: Fact or Fiction?

  - Wills B, Lee SR, Hudson PW, SahraNavard B, de Cesar Netto C, Naranje S, Shah A.Wills B, et al. Foot Ankle Spec. 2019 Feb;12(1):34-38. doi: 10.1177/1938640018762556. Epub 2018 Mar 13.Foot Ankle Spec. 2019. PMID: 29532743

- Shockwave therapy for chronic proximal plantar fasciitis: a meta-analysis.
  - Ogden JA, Alvarez RG, Marlow M.Ogden JA, et al. Foot Ankle Int. 2002 Apr;23(4):301-8. doi: 10.1177/107110070202300402.Foot Ankle Int. 2002. PMID: 11991474

- Operative treatment of subcalcaneal pain.
  - Tountas AA, Fornasier VL.Tountas AA, et al. Clin Orthop Relat Res. 1996 Nov;(332):170-8. doi: 10.1097/00003086-199611000-00023.Clin Orthop Relat Res. 1996. PMID: 8913160

- The role of hamstring tightness in plantar fasciitis.
  - Harty J, Soffe K, O'Toole G, Stephens MM.Harty J, et al. Foot Ankle Int. 2005 Dec;26(12):1089-92. doi: 10.1177/107110070502601215.Foot Ankle Int. 2005. PMID: 16390645

- Plantar calcaneal enthesophytes: new observations regarding sites of origin based on radiographic, MR imaging, anatomic, and paleopathologic analysis.
  - Abreu MR, Chung CB, Mendes L, Mohana-Borges A, Trudell D, Resnick D.Abreu MR, et al. Skeletal Radiol. 2003 Jan;32(1):13-21. doi: 10.1007/s00256-002-0585-x. Epub 2002 Nov 22.Skeletal Radiol. 2003. PMID: 12525939

- [Anatomical variants of the medial calcaneal nerve and the Baxter nerve in the tarsal tunnel].
  - Martín-Oliva X, Elgueta-Grillo J, Veliz-Ayta P, Orosco-Villaseñor S, Elgueta-Grillo M, Viladot-Perice R.Martín-Oliva X, et al. Acta Ortop Mex. 2013 Jan-Feb;27(1):38-42.Acta Ortop Mex. 2013. PMID: 24701749

- [Arthroscopic treatment for calcaneal spur syndrome].
  - Stropek S, Dvorák M.Stropek S, et al. Acta Chir Orthop Traumatol Cech. 2008 Oct;75(5):363-8.Acta Chir Orthop Traumatol Cech. 2008. PMID: 19026190

- Effects of surgical correction for the treatment of adult acquired flatfoot deformity: a computational investigation.
  - Iaquinto JM, Wayne JS.Iaquinto JM, et al. J Orthop Res. 2011 Jul;29(7):1047-54. doi: 10.1002/jor.21379. Epub 2011 Feb 11.J Orthop Res. 2011. PMID: 21319218

- Correlation between the outcome of extracorporeal shockwave therapy and pretreatment MRI findings for chronic plantar fasciitis.
  - Maki M, Ikoma K, Imai K, Kido M, Hara Y, Arai Y, Fujiwara H, Kubo T.Maki M, et al. Mod Rheumatol. 2015 May;25(3):427-30. doi: 10.3109/14397595.2014.978526. Epub 2014 Nov 17.Mod Rheumatol. 2015. PMID: 25401230

- [Sinus tarsi syndrome: what hurts?].
  - Herrmann M, Pieper KS.Herrmann M, et al. Unfallchirurg. 2008 Feb;111(2):132-6. doi: 10.1007/s00113-007-1387-3.Unfallchirurg. 2008. PMID: 18219473

- Bilateral Baxter's neuropathy secondary to plantar fasciitis.
    - Dirim B, Resnick D, Ozenler NK.Dirim B, et al. Med Sci Monit. 2010 Apr;16(4):CS50-53.Med Sci Monit. 2010. PMID: 20357723

- Treatment of chronic haematogenous osteomyelitis of the os calcis.
    - Eid AM.Eid AM. Acta Orthop Scand. 1977;48(6):712-7. doi: 10.3109/17453677708994822.Acta Orthop Scand. 1977. PMID: 607762

- [Aponeurotomy and degenerative lesions of the plantar aponeurosis. Contribution of magnetic imaging].
    - Jardé O, Trinquier-Lautard JL, Boulu G, Grumbach Y, Vives P.Jardé O, et al. Rev Chir Orthop Reparatrice Appar Mot. 1996;82(7):629-35.Rev Chir Orthop Reparatrice Appar Mot. 1996. PMID: 9091981

- Biomechanical evaluation of reconstruction plates with locking, nonlocking, and hybrid screws configurations in calcaneal fracture: a finite element model study.
- Chen CH, Hung C, Hsu YC, Chen CS, Chiang CC.Chen CH, et al. Med Biol Eng Comput. 2017 Oct;55(10):1799-1807. doi: 10.1007/s11517-017-1623-0. Epub 2017 Feb 21.Med Biol Eng Comput. 2017. PMID: 28224272

- Chronic plantar fasciitis: acute changes in the heel after extracorporeal high-energy shock wave therapy--observations at MR imaging.
    - Zhu F, Johnson JE, Hirose CB, Bae KT.Zhu F, et al. Radiology. 2005 Jan;234(1):206-10. doi: 10.1148/radiol.2341031653. Epub 2004 Nov 24.Radiology. 2005. PMID: 15564391

- Endoscopic treatment of calcaneal spur syndrome: A comprehensive technique.
    - Blanco CE, Leon HO, Guthrie TB.Blanco CE, et al. Arthroscopy. 2001 May;17(5):517-22. doi: 10.1053/jars.2001.24065.Arthroscopy. 2001. PMID: 11337720

- Safe Zone for Neural Structures in Medial Displacement Calcaneal Osteotomy: A Cadaveric and Radiographic Investigation.
    - Talusan PG, Cata E, Tan EW, Parks BG, Guyton GP.Talusan PG, et al. Foot Ankle Int. 2015 Dec;36(12):1493-8. doi: 10.1177/1071100715595696. Epub 2015 Jul 31.Foot Ankle Int. 2015. PMID: 26231200

- Segmental foot mobility in individuals with and without diabetes and neuropathy.
    - Rao S, Saltzman C, Yack HJ.Rao S, et al. Clin Biomech (Bristol, Avon). 2007 May;22(4):464-71. doi: 10.1016/j.clinbiomech.2006.11.013. Epub 2007 Feb 22.Clin Biomech (Bristol, Avon). 2007. PMID: 17320257

- Endoscopic Plantar Fasciotomy Improves Early Postoperative Results: A Retrospective Comparison of Outcomes After Endoscopic Versus Open Plantar Fasciotomy.
    - Chou AC, Ng SY, Koo KO.Chou AC, et al. J Foot Ankle Surg. 2016 Jan-Feb;55(1):9-15. doi: 10.1053/j.jfas.2015.02.005. Epub 2015 May 23.J Foot Ankle Surg. 2016. PMID: 26007627

- The medial and inferior calcaneal nerves: an anatomic study.
    - Louisia S, Masquelet AC.Louisia S, et al. Surg Radiol Anat. 1999;21(3):169-73. doi: 10.1007/BF01630895.Surg Radiol Anat. 1999. PMID: 10431329

- Sonographic analysis of enthesopathy in the lower extremities of patients with spondylarthropathy.
  - Lehtinen A, Taavitsainen M, Leirisalo-Repo M.Lehtinen A, et al. Clin Exp Rheumatol. 1994 Mar-Apr;12(2):143-8.Clin Exp Rheumatol. 1994. PMID: 8039281

- Biomechanical consequences of lateral column lengthening of the calcaneus: Part I. Long plantar ligament strain.
  - Dinucci KR, Christensen JC, Dinucci KA.Dinucci KR, et al. J Foot Ankle Surg. 2004 Jan-Feb;43(1):10-5. doi: 10.1053/j.jfas.2003.11.013.J Foot Ankle Surg. 2004. PMID: 14752758

- Extracorporeal Shockwave Therapy Plus Rehabilitation for Patients With Chronic Plantar Fasciitis Might Reduce Pain and Improve Function but Still Not Lead to Increased Activity: A Case-Series Study With Multiple Outcome Measures.
  - Wheeler PC, Tattersall C.Wheeler PC, et al. J Foot Ankle Surg. 2018 Mar-Apr;57(2):339-345. doi: 10.1053/j.jfas.2017.07.001. Epub 2017 Oct 9.J Foot Ankle Surg. 2018. PMID: 29032913

- The novel analgesic, F 13640, produces intra- and postoperative analgesia in a rat model of surgical pain.
  - Kiss I, Degryse AD, Bardin L, Gomez de Segura IA, Colpaert FC.Kiss I, et al. Eur J Pharmacol. 2005 Oct 31;523(1-3):29-39. doi: 10.1016/j.ejphar.2005.09.003. Epub 2005 Oct 13.Eur J Pharmacol. 2005. PMID: 16226246

- The role of bone scintigraphy and plain radiography in intractable plantar fasciitis.
  - Tudor GR, Finlay D, Allen MJ, Belton I.Tudor GR, et al. Nucl Med Commun. 1997 Sep;18(9):853-6. doi: 10.1097/00006231-199709000-00009.Nucl Med Commun. 1997. PMID: 9352552

- The plantar ecchymosis sign in fractures of the calcaneus.
  - Richman JD, Barre PS.Richman JD, et al. Clin Orthop Relat Res. 1986 Jun;(207):122-5.Clin Orthop Relat Res. 1986. PMID: 3720074

- A comparative radiologic examination for unresponsive plantar fasciitis.
  - Kell PM.Kell PM. J Manipulative Physiol Ther. 1994 Jun;17(5):329-34.J Manipulative Physiol Ther. 1994. PMID: 7930967

- Endoscopic decompression of the first branch of the lateral plantar nerve.
  - Lui TH.Lui TH. Arch Orthop Trauma Surg. 2007 Nov;127(9):859-61. doi: 10.1007/s00402-007-0380-1. Epub 2007 Jun 21.Arch Orthop Trauma Surg. 2007. PMID: 17581758

- Extracorporeal shock wave application for chronic plantar fasciitis associated with heel spurs: prediction of outcome by magnetic resonance imaging.
  - Maier M, Steinborn M, Schmitz C, Stäbler A, Köhler S, Pfahler M, Dürr HR, Refior HJ.Maier M, et al. J Rheumatol. 2000 Oct;27(10):2455-62.J Rheumatol. 2000. PMID: 11036844

- Clinical Outcomes After Extracorporeal Shock Wave Therapy for Chronic Plantar Fasciitis in a Predominantly Active Duty Population.
  - Purcell RL, Schroeder IG, Keeling LE, Formby PM, Eckel TT, Shawen SB.Purcell RL, et al. J Foot Ankle Surg. 2018 Jul-Aug;57(4):654-657. doi:

10.1053/j.jfas.2017.11.030. Epub 2018 Apr 2.J Foot Ankle Surg. 2018. PMID: 29622498

- Role of the plantar fascia in digital stabilization. A case report.
  - Pontious J, Flanigan KP, Hillstrom HJ.Pontious J, et al. J Am Podiatr Med Assoc. 1996 Jan;86(1):43-7. doi: 10.7547/87507315-86-1-43.J Am Podiatr Med Assoc. 1996. PMID: 8808324

- Technique for determining when plantar heel pain can be neural in origin.
  - Dellon AL.Dellon AL. Microsurgery. 2008;28(6):403-6. doi: 10.1002/micr.20510.Microsurgery. 2008. PMID: 18623152

- [Reexamination of the communicating branch between the sural and tibial nerves].
  - Sekiya S, Tokita K, Banneheka SK, Suzuki R, Miyawaki M, Chiba S, Kumaki K.Sekiya S, et al. Kaibogaku Zasshi. 2006 Sep;81(3):83-8.Kaibogaku Zasshi. 2006. PMID: 16999334

- Intracorporeal pneumatic shock application for the treatment of chronic plantar fasciitis: a randomized, double blind prospective clinical trial.
  - Dogramaci Y, Kalaci A, Emir A, Yanat AN, Gökçe A.Dogramaci Y, et al. Arch Orthop Trauma Surg. 2010 Apr;130(4):541-6. doi: 10.1007/s00402-009-0947-0. Epub 2009 Aug 11.Arch Orthop Trauma Surg. 2010. PMID: 19669773

- [Tarsal tunnel syndrome. Nerve compression syndrome in the foot].
  - Wolf K, Posel P, Heimkes B, Hierner R, Schweiberer L.Wolf K, et al. Unfallchirurg. 1991 Jun;94(6):291-4.Unfallchirurg. 1991. PMID: 1876851

- Anatomic basis of plantar flap design: clinical applications.
  - Shaw WW, Hidalgo DA.Shaw WW, et al. Plast Reconstr Surg. 1986 Nov;78(5):637-49. doi: 10.1097/00006534-198611000-00012.Plast Reconstr Surg. 1986. PMID: 3763750

- Treatment of plantar fasciitis and calcaneal spurs with the UC-BL shoe insert.
  - Campbell JW, Inman VT.Campbell JW, et al. Clin Orthop Relat Res. 1974;(103):57-62. doi: 10.1097/00003086-197409000-00037.Clin Orthop Relat Res. 1974. PMID: 4416727

- Quantitative scintigraphy in diagnosis and management of plantar fasciitis (calcaneal periostitis): concise communication.
  - Sewell JR, Black CM, Chapman AH, Statham J, Hughes GR, Lavender JP.Sewell JR, et al. J Nucl Med. 1980 Jul;21(7):633-6.J Nucl Med. 1980. PMID: 7391835

- [Plantar fasciotomy in talipes calcaneus. Ceport on 324 surgical operations according to the von Spitzy method].
  - NEUGEBAUER H.NEUGEBAUER H. Arch Orthop Unfallchir. 1961;52:653-8. doi: 10.1007/BF00415410.Arch Orthop Unfallchir. 1961. PMID: 13728509

- Endoscopic release of plantar fasciitis--a benign procedure?
  - Jerosch J.Jerosch J. Foot Ankle Int. 2000 Jun;21(6):511-3. doi: 10.1177/107110070002100611.Foot Ankle Int. 2000. PMID: 10884112

- Magnetic resonance imaging evaluation of calcaneal fat pads in patients with os calcis fractures.

- Levy AS, Berkowitz R, Franklin P, Corbett M, Whitelaw GP.Levy AS, et al. Foot Ankle. 1992 Feb;13(2):57-62. doi: 10.1177/107110079201300202.Foot Ankle. 1992. PMID: 1572588

- Enthesopathy in leprosy.
  - Carpintero-Benítez P, Logroño C, Collantes-Estevez E.Carpintero-Benítez P, et al. J Rheumatol. 1996 Jun;23(6):1020-1.J Rheumatol. 1996. PMID: 8782134

- Medial displacement calcaneal osteotomy reduces the excess forces in the medial longitudinal arch of the flat foot.
  - Arangio GA, Salathé EP.Arangio GA, et al. Clin Biomech (Bristol, Avon). 2001 Jul;16(6):535-9. doi: 10.1016/s0268-0033(01)00011-0.Clin Biomech (Bristol, Avon). 2001. PMID: 11427297

- The deep posterior compartmental syndrome of the leg.
  - Matsen FA 3rd, Clawson DK.Matsen FA 3rd, et al. J Bone Joint Surg Am. 1975 Jan;57(1):34-9.J Bone Joint Surg Am. 1975. PMID: 1123369

- Calcaneal heel spurs: etiology, treatment, and a new surgical approach.
  - Michetti ML, Jacobs SA.Michetti ML, et al. J Foot Surg. 1983 Fall;22(3):234-9.J Foot Surg. 1983. PMID: 6619521

- An uncommon cause of foot pain: the cuboid insufficiency stress fracture.
  - Franco M, Albano L, Kacso I, Gaïd H, Jaeger P.Franco M, et al. Joint Bone Spine. 2005 Jan;72(1):76-8. doi: 10.1016/j.jbspin.2004.03.001.Joint Bone Spine. 2005. PMID: 15681254

- [Plantar release in the treatment of pes cavus in childhood. Technique and indications (author's transl)].
  - Giriat A, Taussig G, Masse P.Giriat A, et al. Rev Chir Orthop Reparatrice Appar Mot. 1979 Mar;65(2):77-86.Rev Chir Orthop Reparatrice Appar Mot. 1979. PMID: 158202

- Chronic plantar fascial inflammation and fibrosis.
  - LeMelle DP, Kisilewicz P, Janis LR.LeMelle DP, et al. Clin Podiatr Med Surg. 1990 Apr;7(2):385-9.Clin Podiatr Med Surg. 1990. PMID: 2346891

- Disorders of the plantar aponeurosis: a spectrum of MR imaging findings.
  - Theodorou DJ, Theodorou SJ, Farooki S, Kakitsubata Y, Resnick D.Theodorou DJ, et al. AJR Am J Roentgenol. 2001 Jan;176(1):97-104. doi: 10.2214/ajr.176.1.1760097.AJR Am J Roentgenol. 2001. PMID: 11133545

- Management of Heel Pad Degloving Injury After Severe Foot Crush Injury: A Case Report Study.
  - Giotis D, Kotsias C, Plakoutsis S, Malahias MA, Konstantinidis C.Giotis D, et al. Cureus. 2021 Mar 30;13(3):e14191. doi: 10.7759/cureus.14191.Cureus. 2021. PMID: 33816037

- Avulsion fracture of the calcaneus at the origin of the abductor hallucis muscle.
  - Pelletier JP, Kanat IO.Pelletier JP, et al. J Foot Surg. 1990 May-Jun;29(3):268-71.J Foot Surg. 1990. PMID: 2380499

- Fatigue perturbation of the os calcis.

        - Smith SD, Young-Paden B, Smith SB, Ellis WN.Smith SD, et al. J Foot Ankle Surg. 1994 Jul-Aug;33(4):402-10.J Foot Ankle Surg. 1994. PMID: 7951194

    - Persistence of enthesopathic changes in patients with spondylarthropathy during a 6-month follow-up.
        - Lehtinen A, Leirisalo-Repo M, Taavitsainen M.Lehtinen A, et al. Clin Exp Rheumatol. 1995 Nov-Dec;13(6):733-6.Clin Exp Rheumatol. 1995. PMID: 8835246

    - Flat foot. A preliminary report of an operation for severe cases.
        - Jones BS.Jones BS. J Bone Joint Surg Br. 1975 Aug;57(3):279-82.J Bone Joint Surg Br. 1975. PMID: 1158938

Search strategy:

(Achilles tendon) and (calcaneus) and (plantar fascia)

Effectiveness of the Simultaneous Stretching of the Achilles Tendon and Plantar Fascia in Individuals With Plantar Fasciitis.
Engkananuwat P, Kanlayanaphotporn R, Purepong N.Foot Ankle Int. 2018 Jan;39(1):75-82. doi: 10.1177/1071100717732762. Epub 2017 Oct 6.PMID: 28985685

A Radiographic Study of Biomechanical Relationship between the Achilles Tendon and Plantar Fascia.
Zhu G, Wang Z, Yuan C, Geng X, Zhang C, Huang J, Wang X, Ma X.Biomed Res Int. 2020 Feb 18;2020:5319640. doi: 10.1155/2020/5319640. eCollection 2020.PMID: 32149113 **Free**

An ossifying bridge - on the structural continuity between the Achilles tendon and the plantar fascia.
Zwirner J, Zhang M, Ondruschka B, Akita K, Hammer N.Sci Rep. 2020 Sep 3;10(1):14523. doi: 10.1038/s41598-020-71316-z.PMID: 32884015

Influence of different knee and ankle ranges of motion on the elasticity of triceps surae muscles, Achilles tendon, and plantar fascia.
Liu CL, Zhou JP, Sun PT, Chen BZ, Zhang J, Tang CZ, Zhang ZJ.Sci Rep. 2020 Apr 20;10(1):6643. doi: 10.1038/s41598-020-63730-0.PMID: 32313166
5

Pes Cavus.
Seaman TJ, Ball TA.2021 May 4. In: StatPearls [Internet]. Treasure Island (FL): StatPearls Publishing; 2021 Jan–.PMID: 32310476 **Free Books & Documents.** Review.

Randomized controlled trial of calcaneal taping, sham taping, and plantar fascia stretching for the short-term management of plantar heel pain.
Hyland MR, Webber-Gaffney A, Cohen L, Lichtman PT.J Orthop Sports Phys Ther. 2006 Jun;36(6):364-71. doi: 10.2519/jospt.2006.2078.PMID: 16776486 Clinical Trial.

Anatomy of the Achilles tendon and plantar fascia in relation to the calcaneus in various age groups.
Snow SW, Bohne WH, DiCarlo E, Chang VK.Foot Ankle Int. 1995 Jul;16(7):418-21. doi: 10.1177/107110079501600707.PMID: 7550955

Ultrasonography Features of the Plantar Fascia Complex in Patients with Chronic Non-Insertional Achilles Tendinopathy: A Case-Control Study.
Romero-Morales C, Martín-Llantino PJ, Calvo-Lobo C, López-López D, Sánchez-Gómez R, De-La-Cruz-Torres B, Rodríguez-Sanz D.Sensors (Basel). 2019 May 2;19(9):2052. doi: 10.3390/s19092052.PMID: 31052554 **Free PMC article.**

Effect of Achilles tendon loading on plantar fascia tension in the standing foot.
Cheung JT, Zhang M, An KN.Clin Biomech (Bristol, Avon). 2006 Feb;21(2):194-203. doi: 10.1016/j.clinbiomech.2005.09.016. Epub 2005 Nov 8.PMID: 16288943

Anatomical variations of the plantar fascia's origin with respect to age and sex-an MRI based study.
Pękala PA, Kaythampillai L, Skinningsrud B, Loukas M, Walocha JA, Tomaszewski KA.Clin Anat. 2019 May;32(4):597-602. doi: 10.1002/ca.23342. Epub 2019 Apr 1.PMID: 30701591

On the morphological relations of the Achilles tendon and plantar fascia via the calcaneus: a cadaveric study.
Singh A, Zwirner J, Templer F, Kieser D, Klima S, Hammer N.Sci Rep. 2021 Mar 16;11(1):5986. doi: 10.1038/s41598-021-85251-0.PMID: 33727610 **Free PMC article.**

Heel spur formation and the subcalcaneal enthesis of the plantar fascia.
Kumai T, Benjamin M.J Rheumatol. 2002 Sep;29(9):1957-64.PMID: 12233893

The Calcaneal Crescent in Patients With and Without Plantar Fasciitis: An Ankle MRI Study.
Finkenstaedt T, Siriwanarangsun P, Statum S, Biswas R, Anderson KE, Bae WC, Chung CB.AJR Am J Roentgenol. 2018 Nov;211(5):1075-1082. doi: 10.2214/AJR.17.19399. Epub 2018 Aug 30.PMID: 30160979

Biomechanical analysis of minimally invasive crossing screw fixation for calcaneal fractures: Implications to early weight-bearing rehabilitation.
Zhang H, Lv ML, Liu Y, Sun W, Niu W, Wong DW, Ni M, Zhang M.Clin Biomech (Bristol, Avon). 2020 Dec;80:105143. doi: 10.1016/j.clinbiomech.2020.105143. Epub 2020 Aug 15.PMID: 32829234

MR Imaging Findings in Heel Pain.
Chang CD, Wu JS.Magn Reson Imaging Clin N Am. 2017 Feb;25(1):79-93. doi: 10.1016/j.mric.2016.08.011.PMID: 27888853 Review.

Ultrasound of entheses in ankylosing spondylitis patients: The importance of the calcaneal and quadriceps entheses for differentiating patients from healthy individuals.
Ishida SN, Furtado RNV, Rosenfeld A, Proglhof JEP, Estrela GBQ, Natour J.Clinics (Sao Paulo). 2019;74:e727. doi: 10.6061/clinics/2019/e727. Epub 2019 Apr 8.PMID: 30970118

The anatomical footprint of the Achilles tendon: a cadaveric study.
Ballal MS, Walker CR, Molloy AP.Bone Joint J. 2014 Oct;96-B(10):1344-8. doi: 10.1302/0301-620X.96B10.33771.PMID: 25274919

The variability of the Achilles tendon insertion: a cadaveric examination.
Kim PJ, Richey JM, Wissman LR, Steinberg JS.J Foot Ankle Surg. 2010 Sep-Oct;49(5):417-20. doi: 10.1053/j.jfas.2010.05.002. Epub 2010 Jun 25.PMID: 20579910

Finite element analysis of plantar fascia during walking: a quasi-static simulation.

Chen YN, Chang CW, Li CT, Chang CH, Lin CF.Foot Ankle Int. 2015 Jan;36(1):90-7. doi: 10.1177/1071100714549189. Epub 2014 Sep 4.PMID: 25189539

Calcaneal Avulsion Fractures: An Often Forgotten Diagnosis.
Yu SM, Yu JS.AJR Am J Roentgenol. 2015 Nov;205(5):1061-7. doi: 10.2214/AJR.14.14190.PMID: 26496554 Review.

Radiological characteristics of the calcaneal spurs in psoriatic arthritis.
Gladman DD, Abufayyah M, Salonen D, Thavaneswaran A, Chandran V.Clin Exp Rheumatol. 2014 May-Jun;32(3):401-3. Epub 2014 May 12.PMID: 24850064 Clinical Trial.

Development of the human Achilles tendon enthesis organ.
Shaw HM, Vázquez OT, McGonagle D, Bydder G, Santer RM, Benjamin M.J Anat. 2008 Dec;213(6):718-24. doi: 10.1111/j.1469-7580.2008.00997.x.PMID: 19094187

The role of biomechanical factors and HLA-B27 in magnetic resonance imaging-determined bone changes in plantar fascia enthesopathy.
McGonagle D, Marzo-Ortega H, O'Connor P, Gibbon W, Pease C, Reece R, Emery P.Arthritis Rheum. 2002 Feb;46(2):489-93. doi: 10.1002/art.10125.PMID: 11840452

Finite element analysis of the foot: Stress and displacement shielding.
Filardi V.J Orthop. 2018 Sep 6;15(4):974-979. doi: 10.1016/j.jor.2018.08.037. eCollection 2018 Dec.PMID: 30224853

Observations on the fibrous retinacula of the heel pad.
Snow SW, Bohne WH.Foot Ankle Int. 2006 Aug;27(8):632-5. doi: 10.1177/107110070602700812.PMID: 16919218

Three-dimensional reconstructions of the Achilles tendon insertion in man.
Milz S, Rufai A, Buettner A, Putz R, Ralphs JR, Benjamin M.J Anat. 2002 Feb;200(Pt 2):145-52. doi: 10.1046/j.0021-8782.2001.00016.x.PMID: 11895112

Development of a Preliminary Ultrasonographic Enthesitis Score in Psoriatic Arthritis - GRAPPA Ultrasound Working Group.
Tom S, Zhong Y, Cook R, Aydin SZ, Kaeley G, Eder L.J Rheumatol. 2019 Apr;46(4):384-390. doi: 10.3899/jrheum.171465. Epub 2018 Oct 15.PMID: 30323008

The contribution of the medial calcaneal osteotomy to the correction of flatfoot deformities.
Nyska M, Parks BG, Chu IT, Myerson MS.Foot Ankle Int. 2001 Apr;22(4):278-82. doi: 10.1177/107110070102200402.PMID: 11354439

Calcaneal osteotomy for the treatment of plantar fasciitis.
Miyamoto W, Takao M, Uchio Y.Arch Orthop Trauma Surg. 2010 Feb;130(2):151-4. doi: 10.1007/s00402-009-0879-8. Epub 2009 Apr 21.PMID: 19381659

Validity of enthesis ultrasound assessment in spondyloarthropathy.
de Miguel E, Cobo T, Muñoz-Fernández S, Naredo E, Usón J, Acebes JC, Andréu JL, Martín-Mola E.Ann Rheum Dis. 2009 Feb;68(2):169-74. doi: 10.1136/ard.2007.084251. Epub 2008 Apr 7.PMID: 18390909

Databank: Livivo
((plantar fascia) AND calcaneus) AND (achilles tendon)
Total:122

Achilles tendon Murphy, G. Andrew [Hrsg.]
(Foot and ankle clinics ; 14,4) 2009

On the morphological relations of the Achilles tendon and plantar fascia via the calcaneus: a cadaveric study.Singh, A / Zwirner, J / Templer, F / Kieser, D / Klima, S / Hammer, N 2021 Volume 11, Issue 1, Page(s) 5986

A Radiographic Study of Biomechanical Relationship between the Achilles Tendon and Plantar Fascia.Zhu, Genrui / Wang, Zhifeng / Yuan, Chengjie / Geng, Xiang / Zhang, Chao / Huang, Jiazhang / Wang, Xu / Ma, Xin 2020 Volume 2020, Page(s) 5319640

An ossifying bridge - on the structural continuity between the Achilles tendon and the plantar fascia.Zwirner, Johann / Zhang, Ming / Ondruschka, Benjamin / Akita, Keichi / Hammer, Niels 2020 Volume 10, Issue 1, Page(s) 14523

Influence of different knee and ankle ranges of motion on the elasticity of triceps surae muscles, Achilles tendon, and plantar fascia.Liu, Chun-Long / Zhou, Ji-Ping / Sun, Peng-Tao / Chen, Bai-Zhen / Zhang, Jun / Tang, Chun-Zhi / Zhang, Zhi-Jie 2020 Volume 10, Issue 1, Page(s) 6643

An ossifying bridge – on the structural continuity between the Achilles tendon and the plantar fasciaJohann Zwirner / Ming Zhang / Benjamin Ondruschka / Keichi Akita / Niels Hammer Scientific Reports, Vol 10, Iss 1, Pp 1- 2020 Volume 10

Ultrasonographic Evaluation of the Femoral Cartilage, Achilles Tendon, and Plantar Fascia in Young Women Wearing High-Heeled Shoes.Mezian, Kamal / Ata, Ayşe M / Kara, Murat / Şahin Onat, Şule / Gürçay, Eda / Çalışkan, Aslı / Simoes, Maria I T / Akıncı, Ayşen / Özçakar, Levent / Franchignoni, Franco PM & R : the journal of injury, function, and rehabilitation 2019 Volume 11, Issue 6, Page(s) 613–618

Influence of different knee and ankle ranges of motion on the elasticity of triceps surae muscles, Achilles tendon, and plantar fasciaChun-Long Liu / Ji-Ping Zhou / Peng-Tao Sun / Bai-Zhen Chen / Jun Zhang / Chun-Zhi Tang / Zhi-Jie Zhang Scientific Reports, Vol 10, Iss 1, Pp 1- 2020 Volume 10

Effectiveness of the Simultaneous Stretching of the Achilles Tendon and Plantar Fascia in Individuals With Plantar Fasciitis.Engkananuwat, Phoomchai / Kanlayanaphotporn, Rotsalai / Purepong, Nithima Foot & ankle international 2017 Volume 39, Issue 1, Page(s) 75–82

Neglected neurogenic clubfoot treated with Achilles tendon lengthening using Z-plasty, total talectomy, and tibiocalcaneal arthrodesis.Oesman, Ihsan / Sari, Chintya Mutiara International journal of surgery case reports 2021 Volume 84, Page(s) 106051

Elasticity of the Achilles Tendon in Individuals With and Without Plantar Fasciitis: A Shear Wave Elastography Study.Pan, Weiyi / Zhou, Jiping / Lin, Yuyi / Zhang, Zhijie / Wang, Yulong

Frontiers in physiology 2021 Volume 12, Page(s) 686631

Ultrasonography Features of the Plantar Fascia Complex in Patients with Chronic Non-Insertional Achilles Tendinopathy: A Case-Control Study.Romero-Morales, Carlos / Martín-Llantino, Pedro Javier / Calvo-Lobo, César / López-López, Daniel / Sánchez-Gómez, Rubén / De-La-Cruz-Torres, Blanca / Rodríguez-Sanz, David Sensors (Basel, Switzerland) 2019 Volume 19, Issue 9

Finite element analysis of plantar fascia during walking: a quasi-static simulation.Chen, Yen-Nien / Chang, Chih-Wei / Li, Chun-Ting / Chang, Chih-Han / Lin, Cheng-Feng Foot & ankle international 2015 Volume 36, Issue 1, Page(s) 90–97

Effect of Achilles tendon loading on plantar fascia tension in the standing foot.Cheung, Jason Tak-Man / Zhang, Ming / An, Kai-Nan Clinical biomechanics (Bristol, Avon) 2006 Volume 21, Issue 2, Page(s) 194–203

Ultrasonographic study of Achilles tendon and plantar fascia in chondrocalcinosis.Falsetti, Paolo / Frediani, Bruno / Acciai, Caterina / Baldi, Fabio / Filippou, Georgios / Prada, Edwin Parra / Sabadini, Luciano / Marcolongo, Roberto The Journal of rheumatology 2004 Volume 31, Issue 11, Page(s) 2242–2250

The anatomical footprint of the Achilles tendon: a cadaveric study.Ballal, M S / Walker, C R / Molloy, A P The bone & joint journal 2014 Volume 96-B, Issue 10, Page(s) 1344–1348

Finite element analysis of plantar fascia under stretch-the relative contribution of windlass mechanism and Achilles tendon force.Cheng, Hsin-Yi Kathy / Lin, Chun-Li / Wang, Hsien-Wen / Chou, Shih-Wei Journal of biomechanics 2008 Volume 41, Issue 9, Page(s) 1937–1944

The variability of the Achilles tendon insertion: a cadaveric examination.Kim, Paul J / Richey, Johanna-Marie / Wissman, Lance R / Steinberg, John S The Journal of foot and ankle surgery : official publication of the American College of Foot and Ankle Surgeons 2010 Volume 49, Issue 5, Page(s) 417–420

Development of the human Achilles tendon enthesis organ.
Shaw, H M / Vázquez, Osorio T / McGonagle, D / Bydder, G / Santer, R M / Benjamin, M Journal of anatomy 2008 Volume 213, Issue 6, Page(s) 718–724

Anatomy of the Achilles tendon and plantar fascia in relation to the calcaneus in various age groups.Snow, S W / Bohne, W H / DiCarlo, E / Chang, V K Foot & ankle international 1995 Volume 16, Issue 7, Page(s) 418–421

Anatomic reconstruction of neglected Achilles tendon rupture with autogenous peroneal longus tendon by EndoButton fixation.Wang, Chih-Chien / Lin, Leou-Chyr / Hsu, Chao-Kuei / Shen, Pei-Hung / Lien, Shiu-Bii / Hwa, Su-Yang / Pan, Ru-Yu / Lee, Chian-Her The Journal of trauma 2009 Volume 67, Issue 5, Page(s) 1109–1112

Heel spur formation and the subcalcaneal enthesis of the plantar fascia.Kumai, Tsukasa / Benjamin, Mike The Journal of rheumatology 2002 Volume 29, Issue 9, Page(s) 1957–1964

Three-dimensional reconstructions of the Achilles tendon insertion in man.Milz, S / Rufai, A / Buettner, A / Putz, R / Ralphs, J R / Benjamin, M Journal of anatomy 2002 Volume 200, Issue Pt 2, Page(s) 145–152

Randomized controlled trial of calcaneal taping, sham taping, and plantar fascia stretching for the short-term management of plantar heel pain.Hyland, Matthew R / Webber-Gaffney, Alisa / Cohen, Lior / Lichtman, P T Steven W The Journal of orthopaedic and sports physical therapy 2006 Volume 36, Issue 6, Page(s) 364–371

Anatomic study suggests that the morphology of the plantaris tendon may be related to Achilles tendonitis.Olewnik, Łukasz / Wysiadecki, Grzegorz / Polguj, Michał / Topol, Mirosław Surgical and radiologic anatomy : SRA 2017 Volume 39, Issue 1, Page(s) 69–75

The role of biomechanical factors and HLA-B27 in magnetic resonance imaging-determined bone changes in plantar fascia enthesopathy.McGonagle, Dennis / Marzo-Ortega, Helena / O'Connor, Philip / Gibbon, Wayne / Pease, Colin / Reece, Richard / Emery, Paul Arthritis and rheumatism 2002 Volume 46, Issue 2, Page(s) 489–493

The plantaris tendon and a potential role in mid-portion Achilles tendinopathy: an observational anatomical study.van Sterkenburg, Maayke N / Kerkhoffs, Gino M M J / Kleipool, Roeland P / Niek van Dijk, Journal of anatomy 2011 Volume 218, Issue 3, Page(s) 336–341

BIOMECHANICAL EFFECTS OF ORTHOTIC WEDGING ON SPORTS AND ACTIVITIES IN PATIENTS WITH PLANTAR FASCIITISBahramizadeh, Mahmood / Tehraninasr, Ali / Froogh, Bijan / Saeedi, Hassan / Vahabkashani, Reza ISBS - Conference Proceedings Archive; 24 International Symposium on Biomechanics in Sports (2006) ; 1999-4168 2007

Avulsion fracture of the calcaneus at the origin of the abductor hallucis muscle.Pelletier, J P / Kanat, I O The Journal of foot surgery 1990 Volume 29, Issue 3, Page(s) 268–271

Biomechanical analysis of minimally invasive crossing screw fixation for calcaneal fractures: Implications to early weight-bearing rehabilitation.Zhang, Haowei / Lv, Miko Lin / Liu, Yin / Sun, Wanju / Niu, Wenxin / Wong, Duo Wai-Chi / Ni, Ming / Zhang, Ming Clinical biomechanics (Bristol, Avon) 2020 Volume 80, Page(s) 105143

The Calcaneal Crescent in Patients With and Without Plantar Fasciitis: An Ankle MRI Study.Finkenstaedt, Tim / Siriwanarangsun, Palanan / Statum, Sheronda / Biswas, Reni / Anderson, Karen E / Bae, Won C / Chung, Christine B AJR. American journal of roentgenology 2018 Volume 211, Issue 5, Page(s) 1075–1082

Ultrasound of entheses in ankylosing spondylitis patients: The importance of the calcaneal and quadriceps entheses for differentiating patients from healthy individuals.Ishida, Suellen Narimatsu / Furtado, Rita Nely Vilar / Rosenfeld, André / Proglhof, Jorge Ernesto Passos / Estrela, Germana Brigida Queiroga / Natour, Jamil Clinics (Sao Paulo, Brazil) 2019 Volume 74, Page(s) e727

Calcaneodynia Marcelo Pires Prado
Einstein, Vol 6, Iss S1, Pp S146-S 2008 Volume 150

Waldman's atlas of diagnostic ultrasound of painful foot and ankle conditionsWaldman, Steven D.

Three-dimensional printed calcaneal prosthesis following total calcanectomy.Imanishi, Jungo / Choong, Peter F M International journal of surgery case reports 2015 Volume 10, Page(s) 83–87

Why heel spurs are traction spurs after all.Zwirner, Johann / Singh, Aqeeda / Templer, Francesca / Ondruschka, Benjamin / Hammer, Niels Scientific reports 2021 Volume 11, Issue 1, Page(s) 13291

Incidence and risk factors of calcaneal enthesophytes in spondyloarthritis and trauma patients.Kamo, Kenta / Yahiro, Ken-Ichiro Modern rheumatology 2016 Volume 26, Issue 4, Page(s) 598–600
d
Calcaneal osteotomy for the treatment of plantar fasciitis.Miyamoto, Wataru / Takao, Masato / Uchio, Yuji Archives of orthopaedic and trauma surgery 2010 Volume 130, Issue 2, Page(s) 151–154

The gastrocnemius Myerson, Mark S. [Hrsg.] (Foot and ankle clinics ; 19,4) 2014

Tachdjian's procedures in pediatric orthopaedics Herring, John Anthony from the Texas Scottish Rite Hospital for Children 2016

Sports medicine conditions Miller, Mark D. return to play ; recognition, treatment, planning 2014

Waldman's comprehensive atlas of diagnostic ultrasound of painful conditionsWaldman, Steven D. 2016

Management of flexible cavovarus foot in patients with Charcot-Marie-Tooth disease: midterm results.Jordà-Gómez, P / Sánchez-Gonzalez, M / Ortega-Yago, A / Navarrete-Faubel, E / Martínez-Garrido, I / Vicent-Carsí, V Revista espanola de cirugia ortopedica y traumatologia 2021

Biomechanical Evaluation of a Cadaveric Flatfoot Model and Lateral Column Lengthening Technique.Heckmann, Nathanael D / Mercer, Jeffrey N / Wang, Lawrence C / McGarry, Michelle H / Ross, Steven D K / Lee, Thay Q The Journal of foot and ankle surgery : official publication of the American College of Foot and Ankle Surgeons 2021

Heel pain Zgonis, Thomas [Hrsg.] (Clinics in podiatric medicine and surgery ; 22,1) 2005

Finite element analysis of the foot: Stress and displacement shielding.Filardi, V Journal of orthopaedics 2018 Volume 15, Issue 4, Page(s) 974–979

Atlas of ultrasound guided musculoskeletal injectionsMalanga, Gerard A. / Mautner, Kenneth R. 2014

A radiologically guided approach to musculoskeletal anatomyTagliafico, Alberto / Martinoli, Carlo 2013

Minimally invasive foot and ankle surgeryBluman, Eric M. / Chiodo, Christopher P. 2016

Campbell's core orthopaedic proceduresCanale, S. T. / Beaty, James H. / Azar, Frederick M. (Expert consult) 2016

Development of a Preliminary Ultrasonographic Enthesitis Score in Psoriatic Arthritis - GRAPPA Ultrasound Working Group.Tom, Stephanie / Zhong, Yujie / Cook, Richard / Aydin,

Sibel Zehra / Kaeley, Gurjit / Eder, Lihi The Journal of rheumatology 2018 Volume 46, Issue 4, Page(s) 384–390

Cartilage Stiffness Effect on Foot Biomechanics of Chinese Bound Foot: A Finite Element Analysis.Zhang, Yan / Awrejcewicz, Jan / Baker, Julien S / Gu, Yaodong Frontiers in physiology 2018 Volume 9, Page(s) 1434

Radiological characteristics of the calcaneal spurs in psoriatic arthritis.
Gladman, D D / Abufayyah, M / Abuffayah, M / Salonen, D / Thavaneswaran, A / Chandran, V Clinical and experimental rheumatology 2014 Volume 32, Issue 3, Page(s) 401–403

MR Imaging Findings in Heel Pain.Chang, Ching-Di / Wu, Jim S Magnetic resonance imaging clinics of North America 2017 Volume 25, Issue 1, Page(s) 79–93

The report on the co-occurrence of two different rare anatomic variations of the plantaris muscle tendon on both sides of an individual.Olewnik, Ł / Wysiadecki, G / Polguj, M / Topol, M Folia morphologica 2016 Volume 76, Issue 2, Page(s) 331–333

The contribution of the medial calcaneal osteotomy to the correction of flatfoot deformities.
Nyska, M / Parks, B G / Chu, I T / Myerson, M S Foot & ankle international 2001 Volume 22, Issue 4, Page(s) 278–282

The Kager's fat pad radiological anatomy revised.Szaro, Paweł / Polaczek, Mateusz / Ciszek, Bogdan Surgical and radiologic anatomy : SRA 2020 Volume 43, Issue 1, Page(s) 79–86

Ankle and Foot Ultrasound: Reliability of Side-to-Side Comparison of Small Anatomic Structures.Rossi, Federica / Zaottini, Federico / Picasso, Riccardo / Martinoli, Carlo / Tagliafico, Alberto Stefano Journal of ultrasound in medicine : official journal of the American Institute of Ultrasound in Medicine 2018 Volume 38, Issue 8, Page(s) 2143–2153

Diagnostic imaging, musculoskeletal traumaBlankenbaker, Donna G. (Diagnostic imaging ; Expert consult) 2016

Clinical Enthesitis in a Prospective Longitudinal Psoriatic Arthritis Cohort: Incidence, Prevalence, Characteristics, and Outcome.Polachek, Ari / Li, Suzanne / Chandran, Vinod / Gladman, Dafna D Arthritis care & research 2017 Volume 69, Issue 11, Page(s) 1685–1691

Operative techniques in foot and ankle surgery
Easley, Mark E. / Wiesel, Sam W. 2010

Diagnosis of heel pain.
Tu, Priscilla / Bytomski, Jeffrey R American family physician 2011 Volume 84, Issue 8, Page(s) 909–916

Disorders of the heel, rearfoot, and ankle
Ranawat, Chitranjan S. / Positano, Rock G. 1999

Orthopedic massage
Lowe, Whitney theory and technique 2009

Book:The foot and ankleKitaoka, Harold B. [Hrsg.] (Master techniques in orthopaedic surgery) 2002

Assessment of enthesis in patients with psoriatic arthritis and fibromyalgia using clinical examination and ultrasound.
Fiorenza, Alessia / Bonitta, Gianluca / Gerratana, Elisabetta / Marino, Francesca / Sarzi-Puttini, Piercarlo / Salaffi, Fausto / Atzeni, Fabiola Clinical and experimental rheumatology 2020 Volume 38 Suppl 123, Issue 1, Page(s) 31–39

A Modified Ponseti Method for the Treatment of Rigid Idiopathic Congenital Clubfoot.
Zhang, Ge / Zhang, Yuan / Li, Ming The Journal of foot and ankle surgery : official publication of the American College of Foot and Ankle Surgeons 2019 Volume 58, Issue 6, Page(s) 1192–1196

Foot and ankle athletic injuries
Baravarian, Bob (Clinics in podiatric medicine and surgery ; 28,1) 2011

Operative techniques in orthopaedic surgery / 4Wiesel, Sam W. [Hrsg.] 2016

Operative techniques in orthopaedic surgery / 1Wiesel, Sam W. [Hrsg.] 2016

Operative techniques in orthopaedic surgery / 2Wiesel, Sam W. [Hrsg.] 2016

Operative techniques in orthopaedic surgery / 3Wiesel, Sam W. [Hrsg.] 2016

Surgery of the foot and ankle / 1Coughlin, Michael J. / Mann, Roger A. / Saltzman, Charles L. 2007

Surgery of the foot and ankle / 2Coughlin, Michael J. / Mann, Roger A. / Saltzman, Charles L. 2007

Sonography of chronic Achilles tendinopathy: a case-control study.Leung, John L Y / Griffith, James F Journal of clinical ultrasound : JCU 2008 Volume 36, Issue 1, Page(s) 27–32

Minimally invasive treatment of the KobyGard system for plantar fasciitis: a retrospective study.
Xu, Hai-lin / Xu, Lei / Zhang, Dian-ying / Fu, Zhong-guo / Wang, Tian-bing / Zhang, Pei-xun / Jiang, Bao-guo Chinese medical journal 2012 Volume 125, Issue 22, Page(s) 3966–3971

Observations on the fibrous retinacula of the heel pad.
Snow, Stephen W / Bohne, Walther H O Foot & ankle international 2006 Volume 27, Issue 8, Page(s) 632–635

American College of Foot and Ankle Surgeons Clinical Consensus Statement: Diagnosis and Treatment of Adult Acquired Infracalcaneal Heel Pain.
Schneider, Harry P / Baca, John M / Carpenter, Brian B / Dayton, Paul D / Fleischer, Adam E / Sachs, Brett D The Journal of foot and ankle surgery : official publication of the American College of Foot and Ankle Surgeons 2017 Volume 57, Issue 2, Page(s) 370–381

Minimally invasive surgery in orthopedicsScuderi, Giles R. [Hrsg.] 2010

The foot and ankleChang, Thomas J. [Hrsg.] 2005

Detection of enthesitis in children with enthesitis-related arthritis: dolorimetry compared to ultrasonography.
Weiss, Pamela F / Chauvin, Nancy A / Klink, Andrew J / Localio, Russell / Feudtner, Chris / Jaramillo, Diego / Colbert, Robert A / Sherry, David D / Keren, Ron Arthritis & rheumatology (Hoboken, N.J.) 2014 Volume 66, Issue 1, Page(s) 218–227

Von der Achillessehnen-Tendinitis bis zur Knochenzyste. Fersenschmerz--was steckt dahinter?Hoberg, M / Gradinger, R / Rudert, M MMW Fortschritte der Medizin 2007 Volume 149, Issue 24, Page(s) 36–9; quiz 40

Clinical application of neuromuscular techniques / 2 : The lower bodyChaitow, Leon / DeLany, Judith 2011

Heel pain-plantar fasciitis and Achilles enthesopathy.Williams, Seth K / Brage, Michael Clinics in sports medicine 2004 Volume 23, Issue 1, Page(s) 123–144

Operative Therapie von Fuß und Sprunggelenk
Jerosch, Jörg [Hrsg.] / Greitemann, Bernhard Fußchirurgie in Klinik und Praxis ; mit 12 Tabellen 2009

Sportverletzungen von A - Z: GesundheitscoachWessinghage, Thomas / Feil, Wolfgang / Ryffel-Hausch, Jacqueline von Schulmedizin bis Naturheilkunde: rasche Heilung, bessere Regeneration, schnell wieder aktiv 2009

Functional reconstruction of the foot and ankle
Hansen, Sigvard T. 2000

Grabb's encyclopedia of flaps / 2 : Upper extremities, torso, pelvis, and lower extremities
Strauch, Berish / Vasconez, Luis O. / Hall-Findlay, Elizabeth J. / Grabb, William C. [Begr.] 2016

Grabb's encyclopedia of flaps / 1 : Head and neck
Strauch, Berish / Vasconez, Luis O. / Hall-Findlay, Elizabeth J. / Grabb, William C. [Begr.] 2016

Validity of enthesis ultrasound assessment in spondyloarthropathy.de Miguel, E / Cobo, T / Muñoz-Fernández, S / Naredo, E / Usón, J / Acebes, J C / Andréu, J L / Martín-Mola, E Annals of the rheumatic diseases 2009 Volume 68, Issue 2, Page(s) 169–174

Heel pain in a young adolescent baseball player.Leri, Jeffrey P Journal of chiropractic medicine 2009 Volume 3, Issue 2, Page(s) 66–68

Characteristics and Course of Enthesitis in a Juvenile Idiopathic Arthritis Inception Cohort.Rumsey, Dax G / Guzman, Jaime / Rosenberg, Alan M / Huber, Adam M / Scuccimarri, Rosie / Shiff, Natalie J / Bruns, Alessandra / Feldman, Brian M / Eurich, Dean T Arthritis care & research 2017 Volume 70, Issue 2, Page(s) 303–308

Enthesitis in an inception cohort of enthesitis-related arthritis.Weiss, Pamela F / Klink, Andrew J / Behrens, Edward M / Sherry, David D / Finkel, Terri H / Feudtner, Chris / Keren, Ron Arthritis care & research 2011 Volume 63, Issue 9, Page(s) 1307–1312

Reconstruction with tenodesis in an adult flatfoot model. A biomechanical evaluation of four methods.

Thordarson, D B / Schmotzer, H / Chon, J The Journal of bone and joint surgery. American volume 1995 Volume 77, Issue 10, Page(s) 1557–1564

Ultrasound detection of entheseal insertions in the foot of patients with spondyloarthropathy. Borman, Pinar / Koparal, Suha / Babaoğlu, Seçil / Bodur, Hatice Clinical rheumatology 2006 Volume 25, Issue 3, Page(s) 373–377

Close to the bone
Legge, David the treatment of musculo-skeletal disorder with acupuncture and other traditional Chinese medicine 1997

Clinical sports medicineBrukner, Peter / Khan, Karim (Sports medicine series) 2007

FußWirth, Carl-Joachim [Hrsg.] / Arnold, Jürgen 45 Tabellen (Orthopädie und Orthopädische Chirurgie) 2002

Correlations among clinical, radiographic, and sonographic scores for enthesitis in ankylosing spondylitis.
Hamdi, Wafa / Chelli-Bouaziz, Mouna / Ahmed, Mohamed Salah / Ghannouchi, Mohamed Mehdi / Kaffel, Dhia / Ladeb, Mohamed Fethi / Kchir, Mohamed Montacer Joint bone spine 2010 Volume 78, Issue 3, Page(s) 270–274

Clinical sports medicine
Brukner, Peter / Khan, Karim (Sports medicine series) 2009

Clinical application of neuromuscular techniques / 2 : Lower body
Chaitow, Leon / DeLany, Judith 2002

Plantar Fasciitis: Diagnosis and Conservative Management.
Gill The Journal of the American Academy of Orthopaedic Surgeons 1997 Volume 5, Issue 2, Page(s) 109–117

Emergency orthopedics
Simon, Robert R. / Koenigsknecht, Steven J.

Diagnosing heel pain in adults.
Aldridge, Tracy American family physician 2004 Volume 70, Issue 2, Page(s) 33 338

Operative treatment of the difficult stage 2 adult acquired flatfoot deformity.
Mosier-LaClair, S /Pomeroy, G / Manoli, A Foot and ankle clinics 2001 Volume 6, Issue 1, Page(s) 95–119

Fußchirurgie
Pisani, Giacomo 1998

Technik und Stellenwert der Arthrosonographie in der rheumatologischen Diagnostik--Teil 3: Sonographie der Sprunggelenke, des Fusses und der Zehen. Schmidt, W A / Hauer, R W / Banzer, D / Bohl-Bühler, M / Braun, J / Mellerowicz, H / Sattler, H / Backhaus, M Zeitschrift fur Rheumatologie 2002 Volume 61, Issue 3, Page(s) 279–290

Manuelle Therapie und komplexe Rehabilitation / 2 : Untere Körperregionen
Streeck, Uwe 2007

Clinical sports medicine

Brukner, Peter / Khan, Karim 2002

Care of the young athlete

Anderson, Steven J. [Hrsg.] 2010

Sonographic analysis of enthesopathy in the lower extremities of patients with spondylarthropathy. Lehtinen, A / Taavitsainen, M / Leirisalo-Repo, M Clinical and experimental rheumatology 1994 Volume 12, Issue 2, Page(s) 143–148

Clinical sports medicine

Brukner, Peter / Khan, Karim 2000

The significance of enthesopathy as a skeletal phenomenon.

Shaibani, A / Workman, R / Rothschild, B M Clinical and experimental rheumatology 1993 Volume 11, Issue 4, Page(s) 399–403

Baraliakos, Xenofon / Wirth, Carl-Joachim [Hrsg.]

vermeiden - erkennen - behandeln ; 179 Tabellen

2010

Praxis der Orthopädie / 2 : Operative Orthopädie

Wirth, Carl-Joachim [Hrsg.] / Jäger, Michael [Begr.]
63 Tabellen 2001

Frühfunktionell konservative und operative Behandlungsmöglichkeiten sowie Rehabilitationsoptionen bei der Achillessehnenruptur Knobloch, K / Thermann, H / Hüfner, T Sportverletzung · Sportschaden 2007 Volume 21, Issue 01, Page(s) 34–40

A short-term follow-up of enthesitis and arthritis in the active phase of juvenile onset spondyloarthropathies.

Burgos-Vargas, R / Pacheco-Tena, C / Vázquez-Mellado, J

Clinical and experimental rheumatology 2002 Volume 20, Issue 5, Page(s) 727–731
Textbook of disorders and injuries of the musculoskeletal system

Salter, Robert Bruce 1999

Treatment of chronic haematogenous osteomyelitis of the os calcis. Eid, A M Acta orthopaedica Scandinavica 1977 Volume 48, Issue 6, Page(s) 712–717

Book ; Conference proceedings:Abstracts

Asian Oceania Conference of Physical and Rehabilitation Medicine <1, 2008, Nanjing>
May 16 - 19, 2008, Nanjing, China (Journal of rehabilitation medicine : Supplement ; 46)
2008

Orthopädie und Traumatologie

Hipp, Erwin [Hrsg.] / Biemer, Edgar

Anatomy and biomechanics of the hindfoot. Perry, J Clinical orthopaedics and related research 1983 , Issue 177, Page(s) 9–15

A short-term follow-up of enthesitis and arthritis in the active phase of juvenile onset spondyloarthropathies. Burgos-Vargas, R / Pacheco-Tena, C / Vázquez-Mellado, J Clinical and experimental rheumatology

Textbook of disorders and injuries of the musculoskeletal system

Salter, Robert Bruce Treatment of chronic haematogenous osteomyelitis of the os calcis. Eid, A M Acta orthopaedica Scandinavica 1977 Volume 48, Issue 6, Page(s) 712–717

Book ; Conference proceedings:Abstracts

Asian Oceania Conference of Physical and Rehabilitation Medicine <1, 2008, Nanjing>

May 16 - 19, 2008, Nanjing, China (Journal of rehabilitation medicine : Supplement ; 46) 2008

Orthopädie und Traumatologie

Hipp, Erwin [Hrsg.] / Biemer, Edgar

40 Tabellen 2003

Anatomy and biomechanics of the hindfoot.

Perry, J Clinical orthopaedics and related research 1983 , Issue 177, Page(s) 9–15

Included Studies:

An ossifying bridge - on the structural continuity between the Achilles tendon and the plantar fascia.
Zwirner J, Zhang M, Ondruschka B, Akita K, Hammer N.Zwirner J, et al. Sci Rep. 2020 Sep 3;10(1):14523. doi: 10.1038/s41598-020-71316-z.Sci Rep. 2020. PMID: 32884015

Anatomical variations of the plantar fascia's origin with respect to age and sex-an MRI based study.
Pękala PA, Kaythampillai L, Skinningsrud B, Loukas M, Walocha JA, Tomaszewski KA.Pękala PA, et al. Clin Anat. 2019 May;32(4):597-602. doi: 10.1002/ca.23342. Epub 2019 Apr 1.Clin Anat. 2019. PMID: 30701591
On the morphological relations of the Achilles tendon and plantar fascia via the calcaneus: a cadaveric study.
Singh A, Zwirner J, Templer F, Kieser D, Klima S, Hammer N.Singh A, et al. Sci Rep. 2021 Mar 16;11(1):5986. doi: 10.1038/s41598-021-85251-0.Sci Rep. 2021. PMID: 33727610
The anatomical footprint of the Achilles tendon: a cadaveric study.
Ballal MS, Walker CR, Molloy AP.Ballal MS, et al. Bone Joint J. 2014 Oct;96-B(10):1344-8. doi: 10.1302/0301-620X.96B10.33771.Bone Joint J. 2014. PMID: 25274919

The variability of the Achilles tendon insertion: a cadaveric examination.
Kim PJ, Richey JM, Wissman LR, Steinberg JS.Kim PJ, et al. J Foot Ankle Surg. 2010 Sep-Oct;49(5):417-20. doi: 10.1053/j.jfas.2010.05.002. Epub 2010 Jun 25.J Foot Ankle Surg. 2010. PMID: 20579910

Development of the human Achilles tendon enthesis organ.
Shaw HM, Vázquez OT, McGonagle D, Bydder G, Santer RM, Benjamin M.Shaw HM, et al. J Anat. 2008 Dec;213(6):718-24. doi: 10.1111/j.1469-7580.2008.00997.x.J Anat. 2008. PMID: 19094187

Development of the human Achilles tendon enthesis organ.
Shaw HM, Vázquez OT, McGonagle D, Bydder G, Santer RM, Benjamin M.Shaw HM, et al. J Anat. 2008 Dec;213(6):718-24. doi: 10.1111/j.1469-7580.2008.00997.x.J Anat. 2008. PMID: 19094187

Three-dimensional reconstructions of the Achilles tendon insertion in man.
Milz S, Rufai A, Buettner A, Putz R, Ralphs JR, Benjamin M.Milz S, et al. J Anat. 2002 Feb;200(Pt 2):145-52. doi: 10.1046/j.0021-8782.2001.00016.x.J Anat. 2002. PMID: 11895112

Anatomy of the Achilles tendon and plantar fascia in relation to the calcaneus in various age groups.
Snow SW, Bohne WH, DiCarlo E, Chang VK.Snow SW, et al. Foot Ankle Int. 1995 Jul;16(7):418-21. doi: 10.1177/107110079501600707.Foot Ankle Int. 1995. PMID: 7550955

Influence of different knee and ankle ranges of motion on the elasticity of triceps surae muscles, Achilles tendon, and plantar fascia.
Liu CL, Zhou JP, Sun PT, Chen BZ, Zhang J, Tang CZ, Zhang ZJ.Liu CL, et al. Sci Rep. 2020 Apr 20;10(1):6643. doi: 10.1038/s41598-020-63730-0.Sci Rep. 2020. PMID: 32313166

Finite element analysis of plantar fascia during walking: a quasi-static simulation.
Chen YN, Chang CW, Li CT, Chang CH, Lin CF.Chen YN, et al. Foot Ankle Int. 2015 Jan;36(1):90-7. doi: 10.1177/1071100714549189. Epub 2014 Sep 4.Foot Ankle Int. 2015. PMID: 25189539

Effect of Achilles tendon loading on plantar fascia tension in the standing foot.
Cheung JT, Zhang M, An KN.Cheung JT, et al. Clin Biomech (Bristol, Avon). 2006 Feb;21(2):194-203. doi: 10.1016/j.clinbiomech.2005.09.016. Epub 2005 Nov 8.Clin Biomech (Bristol, Avon). 2006. PMID: 16288943

Heel spur formation and the subcalcaneal enthesis of the plantar fascia.
Kumai T, Benjamin M.Kumai T, et al. J Rheumatol. 2002 Sep;29(9):1957-64.J Rheumatol. 2002. PMID: 12233893

Observations on the fibrous retinacula of the heel pad.
Snow SW, Bohne WH.Snow SW, et al. Foot Ankle Int. 2006 Aug;27(8):632-5. doi: 10.1177/107110070602700812.Foot Ankle Int. 2006. PMID: 16919218
